# Hydrated Network Interphase with Dynamic Negatively Charged Microregion Enables Ultra-Stable Aqueous Zinc-Ion Batteries

**DOI:** 10.1007/s40820-026-02262-0

**Published:** 2026-06-29

**Authors:** Yin Yang, Xiaofang Wang, Xin Chen, Jia Yao, Daigan Wang, Luyang Ge, Fei Wang, Lin Lv, Li Tao, Hao Wang, Houzhao Wan

**Affiliations:** Hubei Key Laboratory of Micro-Nanoelectronic Materials and Devices, School of Integrated Circuits, Hubei University, Wuhan, 430062 People’s Republic of China

**Keywords:** Zinc-ion battery, Hydrated network interphase (HNI), Dynamic negatively charged microregion (DNCM), Uniform Zn deposition, Triple synergistic mechanism

## Abstract

Aqueous zinc-ion batteries are promising candidates for large-scale energy storage, yet their development is severely hindered by the interfacial instability of zinc anodes. Distinct from strategies employing pre-formed polymers, this work proposes an innovative monomer-induced in situ interface engineering strategy. By leveraging the preferential adsorption of acrylamide monomers on the Zn surface, a locally high-concentration region is created, which subsequently enables the in situ construction of a stable hydrated network interphase (HNI) triggered synergistically by Zn^2+^ and SO_4_^2−^ during electrochemical cycling. The HNI precisely regulates Zn deposition via a triple synergistic mechanism: Lewis acid–base coordination (C = O···Zn^2+^) provides fixed nucleation sites; dynamically anchored SO_4_^2−^ within the interphase forms negatively charged microregions that homogenize Zn^2+^ flux via Coulombic repulsion; and a dense hydrogen-bonding network effectively confines free water and suppresses side reactions. Benefiting from this multifunctional interphase, the Zn//Zn symmetric cell achieves an ultra-long cycling life of 8650 h (over 360 days) at 1 mA cm^−2^ with excellent reproducibility, the Zn//Ti cell delivers a high average Coulombic efficiency of 99.71% at 5 mA cm^−2^. The Zn//I_2_ full cell retains 89.15% of its capacity after 12,000 cycles. This work provides a novel paradigm for interfacial construction toward high-performance zinc metal anodes.

**Supplementary Information:**

The online version contains supplementary material available at 10.1007/s40820-026-02262-0.

## Highlights

- A hydrated network interphase with dynamic negatively charged microregions is constructed in situ via electrochemically triggered Zn^2+^/SO_4_^2–^ synergy, enabling triple synergistic regulation of Zn deposition.
- The HNI-modified Zn anode achieves ultra-long cycling (8650 h at 1 mA cm^–2^ and 1600 h at 10 mA cm^–2^) and high Coulombic efficiency (99.71%).
- The Zn//I_2_ cell delivers a high specific capacity of 356.27 mAh g^–1^ at 1 A g^–1^ and retains 89.15% capacity after 12,000 cycles.

**Supplementary Information:**

The online version contains supplementary material available at 10.1007/s40820-026-02262-0.

## Graphical abstract

**Figure:**
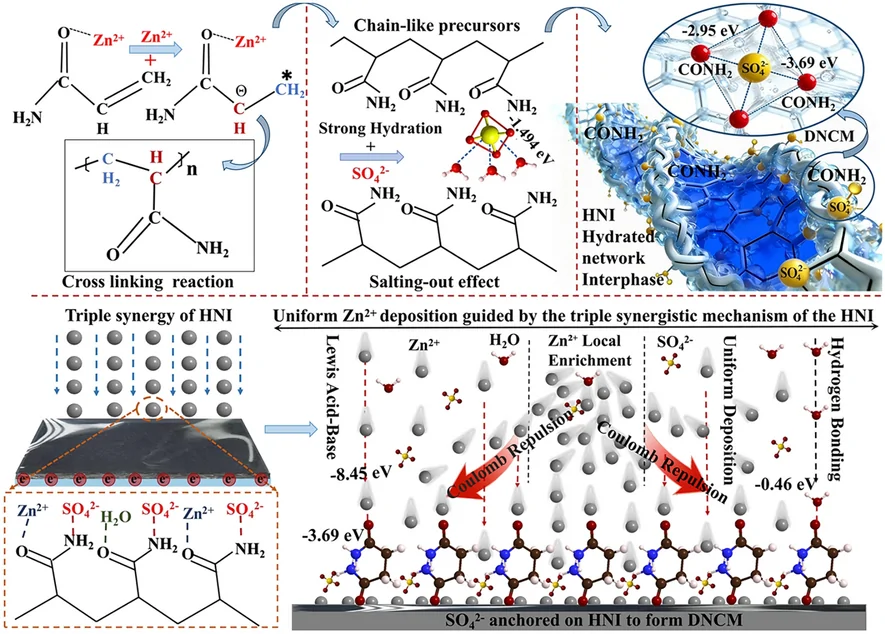

## Introduction

Aqueous zinc-ion batteries (AZIBs) have emerged as promising candidates for grid-scale energy storage [1–9], leveraging the inherent advantages of metallic zinc, including its suitable redox potential (−0.76 V vs. SHE), high reversibility, environmental benignity, and cost-effectiveness [11–17]. However, the practical application of AZIBs is impeded by critical challenges at the Zn anode, such as dendrite growth, corrosion, and hydrogen evolution reactions, which stem from the high reactivity of zinc and water decomposition in aqueous electrolytes [18–21]. The numerous optimization strategies including three-dimensional structural design [22–25], alloying [26], surface/interface engineering [27–34], and electrolyte optimization [35–41] all have been proven to improve the overall performance of zinc anodes [42–46].

Conventional strategies, such as artificial interfacial layers and polymer electrolytes, often face limitations such as complex fabrication [42, 47], poor interfacial contact, and insufficient ionic conductivity [48, 49]. Hydrogel electrolytes, despite offering high ionic conductivity and flexibility for uniform ion transport [50–52], face practical hurdles such as complex fabrication and inadequate interfacial contact [53]. A promising synergistic approach, therefore, is to develop an in situ hydrated network interphase that merges interface engineering with electrolyte modification, combining high conductivity with a seamless electrode interface to overcome these limitations. This can be realized using acrylamide (AM), a polar monomer featuring both acryloyl and amino groups, which exhibits high hydrophilicity and reactivity. Unlike its polymerized form (PAM)—a known corrosion inhibitor that chemisorbs on metal surfaces and suppresses HER—the monomeric AM does not increase solution viscosity, thereby preserving conductivity and wettability [54–56]. Furthermore, AM can be triggered by specific ions to undergo in situ polymerization, offering a practical pathway for interface control [57, 58].

In this work, a stable hydrated network interphase (HNI) enriched with dynamically distributed negatively charged microregions (DNCM) was constructed in situ on the Zn anode. The formation of this functional interphase is based on a unique synergistic triggering mechanism: During electrochemical cycling, the adsorbed AM molecules on the anode surface undergo in situ polymerization, driven by the concerted effects of Zn^2+^ cross-linking and SO_4_^2−^ salting-out, leading to the precise construction of this interface. This in situ formed interphase concurrently addresses the key issues of Zn deposition guidance, ion flux homogenization, and side reaction suppression through a triple synergistic mechanism involving Lewis acid–base coordination, Coulombic repulsion from dynamically anchored DNCM, and a hydrogen-bonding network. Consequently, the Zn metal anode achieves exceptional cycling stability and high reversibility, accompanied by significantly enhanced full-cell performance. This work not only reports a high-performance interphase but also provides a novel paradigm for the precise in situ construction of electrode–electrolyte interfaces through molecular design.

## Experimental Section

### Cathode Preparation

The I2-based cathode material was prepared by mixing activated carbon, commercial acetylene black, and binder in a mass ratio of 8:1:1. The blended powders were thoroughly ground in an agate mortar followed by addition of an appropriate amount of N-methylpyrrolidone (NMP) dispersant. After homogenization through extended grinding, the resultant slurry was uniformly coated onto a hydrophobic carbon paper substrate using a doctor blade. The electrode was dried at 60 °C for solvent evaporation and subsequently impregnated with 3–4 droplets (30 μL/drop) of 2 M ZnI_2_ aqueous solution on an 80 °C hotplate. Following complete solution absorption, thermal treatment was performed at 120 °C for 5 min to finalize the cathode fabrication, maintaining a 1:1 mass ratio between activated carbon and iodine. The processed electrodes were precision-cut into circular disks with an area of 1.13 cm^2^ using a microtome for cell assembly.

### Cell Configuration

The electrochemical cells were assembled in CR2032 coin-type configuration employing either commercial zinc foil (0.15–0.25 mm thickness) or custom-prepared 3D zinc anode as the negative electrode. A glass fiber membrane served as the separator, with all components being meticulously stacked under controlled atmospheric conditions.

## Results and Discussion

To construct a hydrated network interphase with high ionic conductivity and superior interfacial wettability, we employ AM—a polar molecule containing acryloyl and amino groups—as an electrolyte additive.

**Fig. 1:**
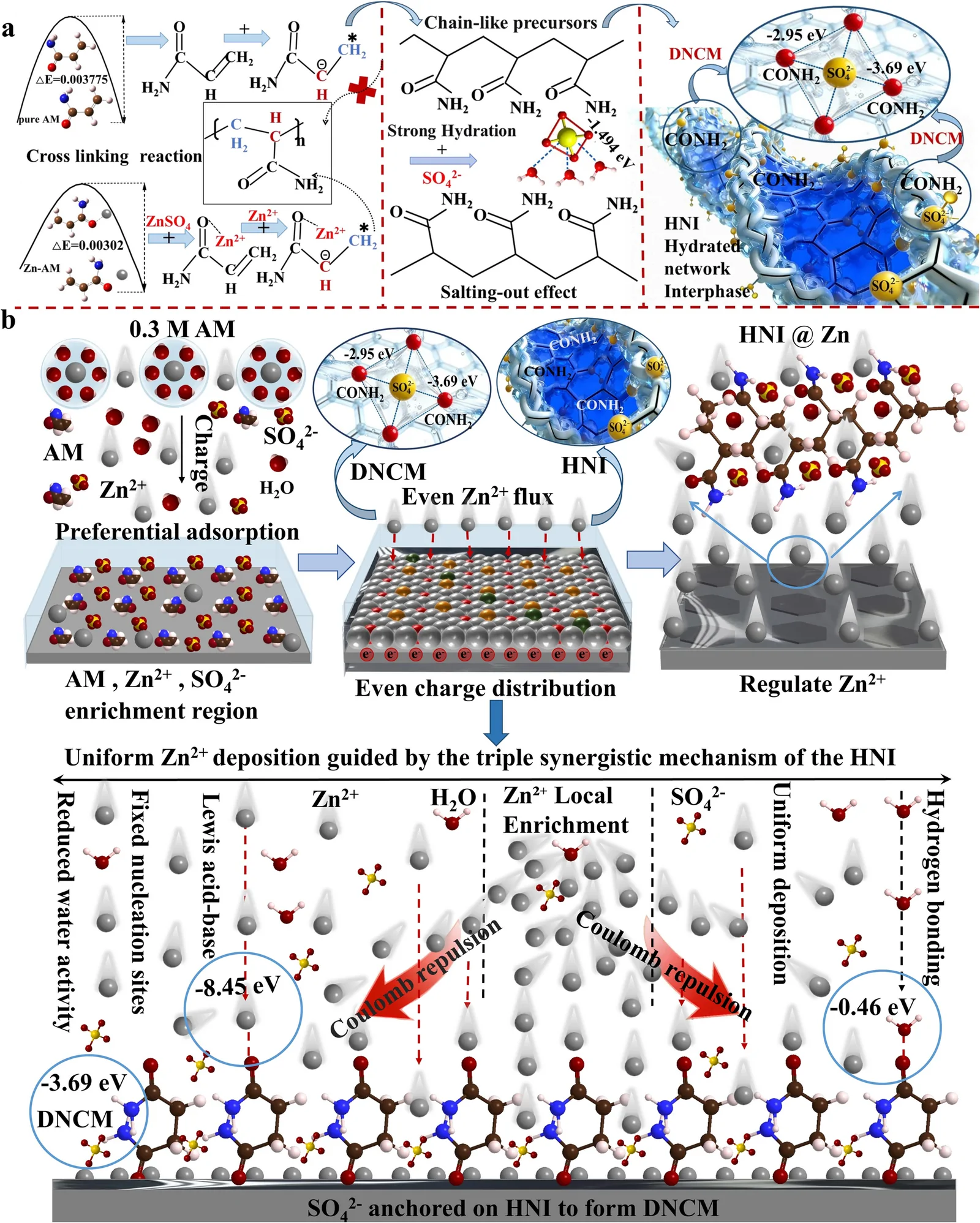
Design Concept and Working Mechanism of the HNI.** a** Schematic diagram of the formation of the hydrate network interface (HNI). **b** Schematic illustration of the Zn deposition process at the electrode interface in 0.3 M AM electrolyte

Driven by its zincophilicity, AM preferentially adsorbs onto the Zn anode surface, creating a localized high-concentration region. Subsequently, Zn^2+^ ions from the electrolyte act as cross-linkers, inducing the polymerization of AM into chain-like precursors. These chains then undergo contraction and aggregation via the salting-out effect of SO_4_^2−^ anions, leading to their spontaneous self-assembly into a stable HNI on the Zn surface. Crucially, this entire assembly process is dramatically intensified during electrochemical cycling by the sustained migration of Zn^2+^ toward the anode and the concomitant enrichment of SO_4_^2−^ within the electrical double layer at the interface. This method requires no additional agents, as the electrolyte’s inherent ions, dynamically recruited by the cycling process, serve as the precise constructors of the interphase (Fig. 1a). Electrostatically anchored within the HNI, SO_4_^2−^ forms a dynamic, hydrated framework enriched with DNCM. The continuous ion migration during cycling ensures the dynamic stability and functional renewal of these DNCM (Fig. S1). This framework alters the apparent solvation sheath of Zn^2+^ via a triple synergistic mechanism, guiding uniform zinc deposition: (1) Lewis acid–base coordination (C = O···Zn^2+^) provides fixed nucleation sites; (2) DNCM homogenizes Zn^2+^ flux via long-range Coulombic repulsion, preventing concentration polarization; and (3) a hydrogen-bonding network (C = O···H–O–H) reduces free water activity, suppressing the hydrogen evolution reaction (HER) [59, 60]. These effects collectively reshape the solvation structure, establish ordered Zn^2+^ transport channels, optimize the electric field distribution, and guide uniform Zn deposition (Fig. 1b). In contrast, in the ZnSO_4_ electrolyte without AM, the Zn anode suffers from severe side reactions—including rampant dendrite growth, hydrogen evolution, and corrosion—which drastically degrade the cell's cycling life (Fig. S2).

### In situ Formation Mechanism and Verification of the HNI

**Fig. 2:**
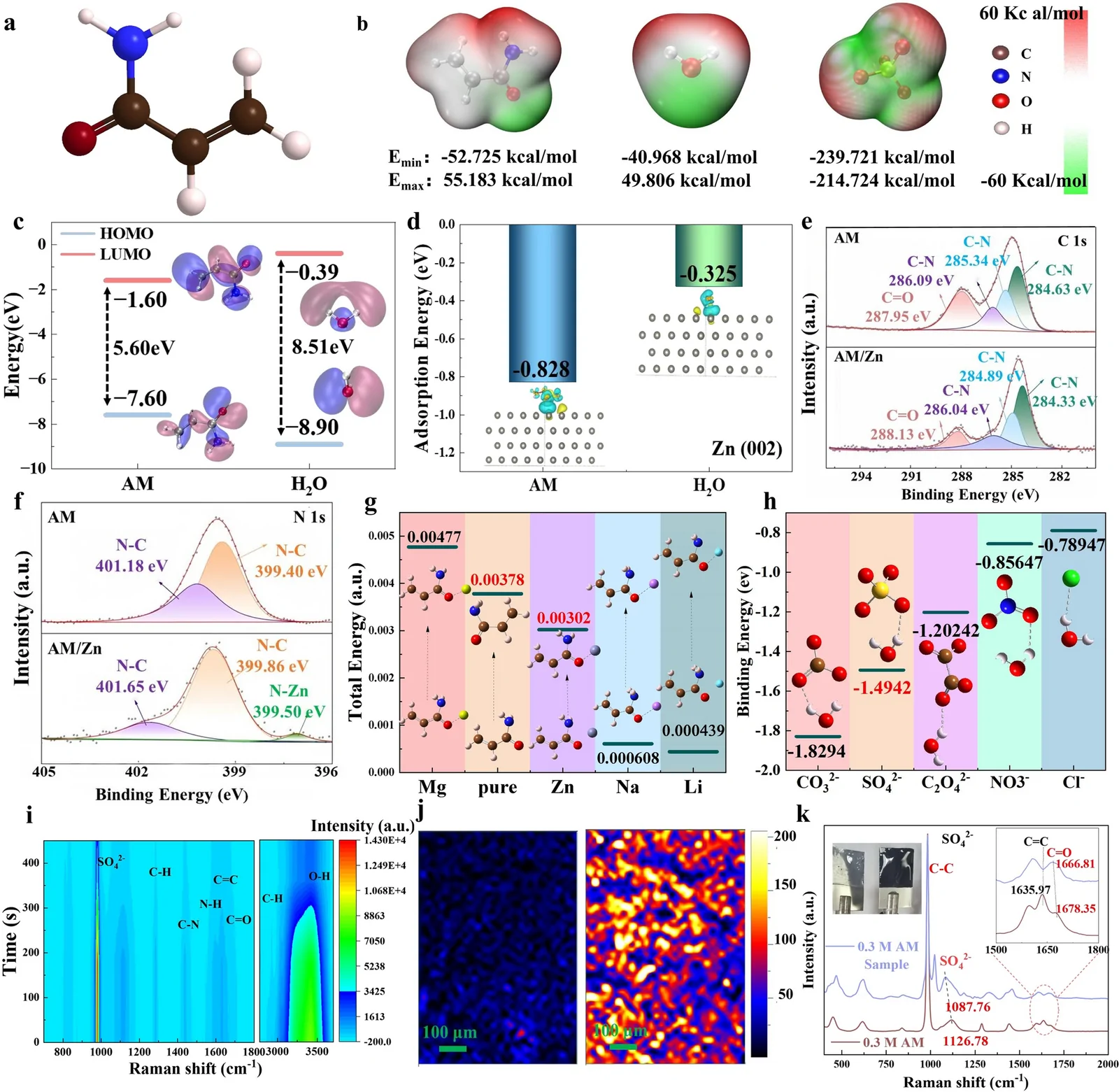
Mechanism of HNI Formation in situ. **a** Molecular structure of the AM. **b** Electrostatic potential mapping of AM, H_2_O and SO_4_^2−^. **c** HOMO and LUMO energy levels for H_2_O and AM. **d** Adsorption energy of H_2_O and AM on the Zn (002) surface. **e** O 1*s* region in XPS spectra of AM powder (top) and dry Zn plate after immersion in AM aqueous solution (bottom). **f** C 1*s* region in XPS spectra of AM powder (top) and dry Zn plate after immersion in AM aqueous solution (bottom). **g** Energy barrier of AM polymerization in the presence or absence of metal cations. **h** Binding energy between anions and water during the anion-induced salting-out effect. **i** In situ Raman contour plot of 0.3 M AM electrolyte. **j** Corresponding Raman mapping images (C-N signal of AM) of the Zn/electrolyte interface at the pristine state (left) and discharge state at 5 mA cm^−2^ (right). **k** Raman characterization of 0.3 M AM and the HNI product formed on Zn surface

The chemical structure of AM reveals the presence of a polar amide group (Fig. 2a). The electrostatic potential (ESP) map in Fig. 2b clearly reveals that the O atom in the -CONH_2_ group of AM exhibits a significantly higher negative charge density compared to water molecules, enabling preferential adsorption on the Zn surface and providing strong zincophilic sites for Zn^2+^ [61]. This promotes solvation structure reorganization and regulates homogeneous Zn^2+^ flux [42, 62]. Furthermore, according to the molecular orbital theory, a narrower band gap between the lowest unoccupied molecular orbital (LUMO) and highest occupied molecular orbital (HOMO) energy levels, as well as a lower LUMO level, indicates an enhanced electron transfer capability, which can lead to improved adsorption of solvent molecules on Zn anodes. Therefore, we conducted molecular orbital level calculations to further investigate the zincophilic property of AM. Figure 2c displays the LUMO and HOMO level of AM and H_2_O, accompanied by their respective frontier molecular orbital diagrams. It is noteworthy that AM exhibits a narrower LUMO–HOMO bandgap (5.60 eV) and lower LUMO energy level (−1.60 eV) compared to H_2_O (8.51 eV), indicating enhanced electron transfer capability and stronger adsorption affinity to the Zn anode [63–65]. DFT calculations further confirmed the stronger interaction between AM and the Zn surface, with adsorption energies of AM on the (002), (101), and (100) planes being substantially lower than those of water (e.g., −0.828 vs. −0.325 eV on (002)), indicating effective displacement of water molecules from the Zn surface (Fig. 2d). The O atom in –CONH_2_ serves as the primary binding site, as evidenced by charge density analysis (Fig. 2d inset). This preferential adsorption reduces side reactions and suppresses the tip effect [66], with consistent trends observed across all major Zn crystal planes (Fig. S3). This electronic structure enables AM to preferentially adsorb on the Zn surface over water molecules [67, 68]. These computational results consistently demonstrate the superior zincophilic nature of AM over H_2_O, predicting its preferential adsorption and subsequent interfacial enrichment on the Zn anode surface. This interfacial enrichment creates a localized high-concentration region of AM, which is essential for the subsequent in situ reactions.

To provide experimental support for the aforementioned analysis, we conducted an immersion test by soaking a zinc foil in an aqueous AM solution. XPS analysis confirmed the adsorption of AM on the zinc anode, with characteristic peaks of C = O (288.13 eV) and C-N (401.65 eV), N–H (399.86 eV) observed in the C 1*s* (Fig. 2e) and N 1*s* (Fig. 2f) spectra. The presence of a Zn-N peak (399.50 eV) and the attenuated C = O signal suggest coordination between the amide groups and the Zn surface. SEM images further demonstrated AM's protective role: After 1 month of immersion, the Zn foil in AM solution remained smooth, whereas severe corrosion occurred in pure water (Fig. S4). The static experiment not only verifies the spontaneous and stable adsorption of AM on Zn, in agreement with calculations, but also provides visual evidence of its efficacy in inhibiting anode degradation.

The specificity of this adsorption and its role as a prerequisite for polymerization were further probed by immersing different metal foils (e.g., Cu, Zn, and Ti) in the AM-containing electrolyte. Only on the Zn foil surface, a sparse, fibrous polymer was occasionally observed after prolonged immersion, while other metals remained clean (Fig. S5). This observation directly corroborates AM’s zincophilic nature. Crucially, even on Zn, this static polymerization was minimal—a stark contrast to the uniform HNI formed during actual electrochemical cycling. This contrast reveals that adsorption alone, while creating a local monomer reservoir, is insufficient to drive complete HNI formation. What are the essential electrochemical conditions that enable this transformation? To answer this, we systematically varied the electrolyte composition during galvanostatic cycling and examined the resulting electrode surfaces. We first compared the two simplest scenarios: Zn cycling in pure AM aqueous solution (lacking both Zn^2+^ and SO_4_^2−^) and in the complete AM/ZnSO_4_ electrolyte. Strikingly, only the latter produced the characteristic transparent HNI (Fig. S6b vs. S6c), immediately suggesting that electrolyte ions are critically involved. The results were remarkably clear: HNI formation occurred only when both Zn^2+^ and SO_4_^2−^ were simultaneously present (Fig. S6 and Table S1). In electrolytes containing Zn^2+^ but lacking SO_4_^2−^ (Zn(NO_3_)_2_, ZnCl_2_), no visible HNI was observed (Fig. S6g, h), indicating that while Zn^2+^ can initiate polymerization, the subsequent assembly into a structured interphase requires SO_4_^2−^. Conversely, in electrolytes containing SO_4_^2−^ but lacking Zn^2+^ (MgSO_4_, Li_2_SO_4_), white flocculent precipitates appeared (Fig. S6d, e), attributed to SO_4_^2−^-induced salting-out of AM without chemical cross-linking. In all other combinations lacking both ions—whether LiNO_3_ or pure AM aqueous solution—no HNI formed (Fig. S6c, f). These systematic controls lead to an unambiguous conclusion: The formation of a functional HNI requires the synergistic action of both Zn^2^⁺ and SO₄^2^⁻ during electrochemical cycling. The HNI formation was effective even under high-current plating conditions (10 mA cm⁻^2^, Fig. S7), demonstrating the robustness of this process.

However, the AM/ZnSO_4_ mixture remained a transparent liquid even after 3 months (Fig. S8), confirming that the HNI forms only as a thin interfacial film during cycling rather than a bulk gel, thus preserving ion transport and electrolyte activity. The confinement of the reaction to the interface is explained by molecular dynamics (MD) simulations, which show that in the bulk electrolyte, AM molecules neither enter the primary solvation shell of Zn^2+^ nor form stable complexes with SO_4_^2−^ (Fig. S9). Raman spectroscopy on electrolytes with varying AM concentrations further confirmed that AM dissolves without perturbing the bulk solvation structure (Fig. S10). Therefore, the decisive trigger is the electrochemically driven local ion enrichment during cycling. The electric field directs a sustained flux of Zn^2+^ toward the anode, with SO_4_^2−^ migrating concomitantly, creating a dramatic local concentration increase of both ions within the pre-adsorbed AM layer. Consequently, the reaction is intrinsically an interfacial event, mandated by the synergy between AM adsorption (creating a local monomer reservoir) and electrochemically driven local ion enrichment (providing the high-concentration trigger and structural agent).

This enriched microenvironment enables a two-step formation mechanism. In general, AM polymerization is not spontaneous and requires an external trigger. DFT calculations first reveal a general cation effect: certain metal cations (e.g., Zn^2+^, Na^+^, and Li^+^) can significantly promote AM polymerization by reducing the energy barrier, with Zn^2+^ exhibiting the strongest binding affinity, while Mg^2+^ conversely impedes the process (as shown in Figs. 2g and S11). Within our specific system, the electrochemically enriched Zn^2+^ plays a pivotal dual role. The oxygen atoms on AM carry substantial negative charge, forming strong interactions with Zn^2+^ and adsorbing it onto the AM surface. Due to its low electronegativity, the adsorbed Zn^2+^ can polarize and extract electrons from the unsaturated C = C bonds, enhancing conjugated electron delocalization. This leads to the cleavage of the C = C double bonds, generating radical intermediates. Subsequently, under the electrochemical reduction conditions, these intermediates initiate chain polymerization, forming the long-chain polymeric precursors of the hydrated interphase [69]. Beyond the cationic trigger, the co-enriched anion dictates the assembly outcome. Extensive studies have shown that chaotropic Hofmeister ions (e.g., CO_3_^2−^, SO_4_^2−^, Cl^−^, NO_3_^−^, and I^−^) can induce a “salting-out” effect. Among them, the sulfate ion (SO_4_^2−^), which is inherently present in our ZnSO_4_ electrolyte, demonstrates exceptional hydration ability (Fig. 2h). When enriched at the interface, SO_4_^2−^ competes for water molecules around the nascent polymer chains, reduces the free water content, and disrupts their hydration shells. This dehydration strengthens hydrophobic interactions, triggering chain contraction and transforming the dispersed precursors into a compact, stable HNI network on the zinc anode surface [70].

The proposed mechanism of electrochemically triggered interfacial polymerization and assembly was directly corroborated by in situ Raman spectroscopy. Mapping of the Zn anode/electrolyte interface at the initial stage (Fig. 2i) revealed characteristic signals of AM monomers (e.g., C = C at 1628.95 cm^−1^ and = CH_2_ at 3055.60 cm^−1^) and electrolyte components (SO_4_^2−^ at 975.42 cm^−1^, broad O–H band) [71]. As plating proceeded, a significant increase in the N–H signal intensity at the interface (Fig. 2j) provided direct spectral evidence for the dynamic adsorption and enrichment of AM molecules [39], creating the local monomer reservoir critical for HNI formation. Concurrently, the rapid diminution of the C = C peak (tracked in Fig. S12) confirmed the triggered polymerization within this enriched layer, while the decrease and redshift of SO_4_^2−^ and O–H signals indicated the incorporation of sulfate and the expulsion of free water into/from the interphase [72], consistent with the predicted “salting-out” effect. Ex situ Raman analysis of the formed HNI (Fig. 2k) yielded further structural evidence: The complete disappearance of the C = C vibration and the emergence of a new C–C peak (1025 cm^−1^) confirmed the alkene hydration reaction; the redshift of C = O stretching (1678 → 1667 cm^−1^) indicated coordination with Zn^2+^ and H_2_O; and the shifted SO_4_^2−^ asymmetric stretching (1127 → 1088 cm^−1^) verified its hydrogen-bonding interaction with the polymer amide groups. In summary, the combination of interfacial spectroscopy and the preceding experiments unequivocally demonstrates that the functional HNI is not a bulk precipitate but is formed via a precisely orchestrated, electrochemically triggered process. This process hinges on the cyclical enrichment of AM, Zn^2+^, and SO_4_^2−^ at the anode interface, where Zn^2+^ catalyzes monomer polymerization, and SO_4_^2−^ directs the assembly of the resulting chains into a compact, stable interphase.

### Interfacial Properties and Triple Synergistic Regulation

**Fig. 3:**
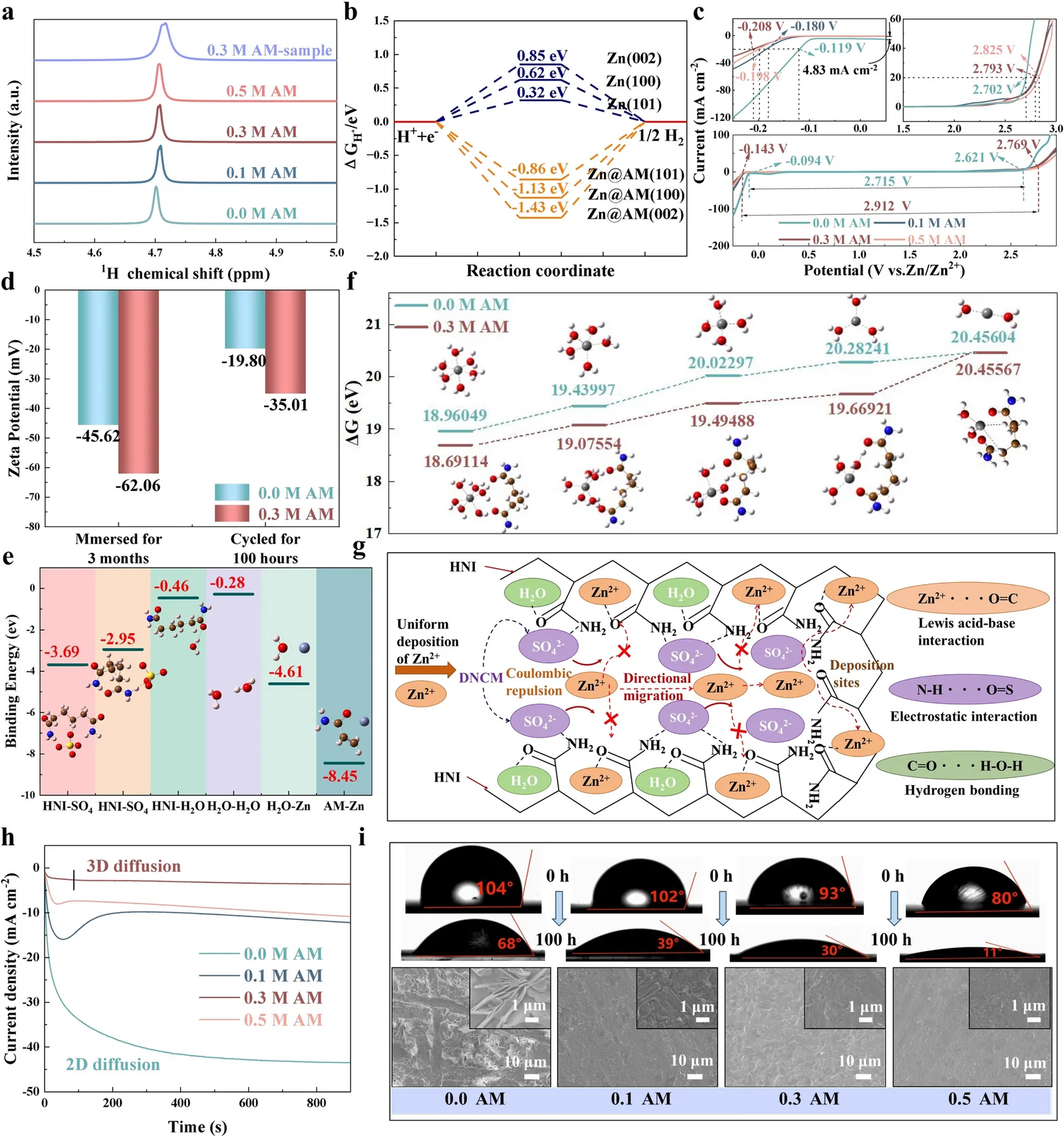
Interfacial Modification and Regulation by HNI. **a** ^1^H NMR spectra of AM electrolytes with different concentrations and their corresponding HNI products on Zn anode. **b** Hydrogen evolution energy barriers on bare Zn and AM-adsorbed Zn anode surfaces. **c** Electrochemical window test at a scan rate of 1 mV s^−1^ within −0.25 ~ 3 V, with magnified hydrogen evolution potential (top-left inset) and oxygen evolution potential (top-right inset). **d** Zeta potential of the Zn sheet surface soaked in 0.0 M AM and 0.3 M AM electrolytes for 3 months, as well as the Zn anode surface after cycling for 100h. **e** Binding energies between various components calculated by first-principles methods. **f** Desolvation energy during Zn^2+^ deposition. **g** Mechanism schematic of HNI-guided uniform Zn^2+^ plating. **h** Chronoamperometry curves of Zn electrodeposition at −150 mV for electrolytes with different AM concentrations. **i** Contact angles and corresponding surface morphologies (SEM) of Zn anodes before and after 100 h cycling in electrolytes with varying AM concentrations

To elucidate the modifying role of the in situ formed HNI, we first assessed its impact on the bulk electrolyte properties. Viscosity and ionic conductivity tests (Fig. S13) confirmed that introducing AM (0.1–0.5 M) into 2 M ZnSO_4_ electrolyte induces only a marginal increase in viscosity and a slight decrease in conductivity, consistent with the dissolution of an organic additive. Crucially, the conductivity remains comparable to that of the pure ZnSO_4_ electrolyte, indicating that the HNI preserves efficient bulk Zn^2+^ migration. These moderate changes confirm that AM primarily functions as an interfacial modifier rather than drastically altering bulk ion transport. This optimal balance between maintained ionic mobility and introduced molecular functionality sets the stage for investigating its profound and dynamic interfacial effects, which are key to stabilizing zinc deposition. ^1^H NMR analysis (Fig. 3a) indicates that while AM addition does not alter the bulk electrolyte structure, a shift in the water signal (4.7 → 4.75 ppm) in the scraped surface sample confirms reduced water activity at the interface. This is attributed to hydrogen bonding (H_2_O···O = C) with the HNI chains, which lowers both the concentration and activity of free water, thereby suppressing its participation in the reduction reaction (H_2_O + e^−^ → ½H_2_ + OH^−^) [73]. Density functional theory (DFT) calculations (Fig. 3b) directly show that HER activation energy is significantly increased on AM-adsorbed Zn surfaces across different facets (e.g., by 68%–82%, see reaction models in Fig. S14), due to this hydrogen-bond network. Electrochemical stability window tests (Fig. 3c) further verify the suppression effect. The onset potential for HER shifts negatively by ~ 49 mV, and the overall stability window expands by approximately 0.2 V, effectively mitigating water decomposition. These findings collectively suggest that AM can reconstruct the Zn anode's solvation structure and reduce active water at the interface, thereby suppressing HER and related side reactions [63, 74].

The electrostatic landscape at the interface is dynamically regulated by the HNI. Zeta potential analysis (Fig. 3d) confirms the formation of SO_4_^2−^-enriched, DNCM within the AM-modified system. The surface potential shifted markedly to more negative values after cycling or long-term immersion (e.g., from −19.80 to −35.01 mV after 100h cycling). This demonstrates the stable anchoring of SO_4_^2−^ within the HNI, which is consistent with its extremely negative electrostatic potential (ESP) as shown in Fig. 2b (E_min_: −239.721 kcal mol^−1^). This strong negative character enables SO_4_^2−^ to electrostatically interact with the locally electropositive -NH_2_ groups of AM, thereby becoming firmly anchored within the HNI to form the DNCM. The resulting DNCM implements the second synergistic mechanism: They homogenize Zn^2+^ distribution via long-range Coulombic repulsion to prevent localized concentration polarization, while the structured interphase confines ion migration pathways, synergistically ensuring uniform deposition. At the core of uniform deposition are the guided desolvation and preferential nucleation processes, rooted in specific interfacial interactions. Binding energy calculations (Fig. 3e) provide atomic-scale insights: The interaction between the amide group (-CONH_2_) and Zn^2+^ is significantly stronger (−8.45 eV) than that between H_2_O and Zn^2+^ (−4.61 eV). This strong Lewis acid–base interaction provides fixed, preferential nucleation sites, directly promoting Zn^2+^ deposition guidance. Moreover, the -CONH_2_···H_2_O interaction (−0.46 eV) exceeds that of H_2_O···H_2_O (−0.28 eV), indicating that free water preferentially binds to the HNI over forming clusters. This mechanism improves interfacial wettability and helps suppress parasitic reactions. The calculations further confirm the anchoring of SO_4_^2−^ within the HNI, revealing two distinct binding sites at −3.69 and −2.95 eV, which is consistent with the formation of the DNCM. These interactions collectively enhance Zn^2+^ mobility and facilitate uniform deposition [75]. 

Building upon these favorable interactions, HNI significantly optimizes the Zn^2+^ desolvation kinetics. As shown in Fig. 3f, the polar amide groups compete with water molecules in the primary solvation shell, while SO_4_^2−^ anions are guided into the coordination sphere to form a transitional [Zn(H_2_O)_4_(SO_4_)] complex. This restructuring markedly reduces the energy barrier for water molecule desorption, thereby facilitating desolvation and enhancing interfacial kinetics. These synergistic interactions are unified in the proposed mechanism (Fig. 3g). The in situ formed HNI reconstructs the interfacial microenvironment via a triple synergistic mechanism: Initially, Lewis acid–base coordination (Zn^2+^···C = O) guides preferential Zn^2+^ deposition at “seed sites”; as deposition proceeds, DNCM homogenizes ion flux via Coulombic repulsion, preventing concentration polarization and ensuring ordered growth; simultaneously, hydrogen-bonded water molecules (C = O···H–O–H) enhance electrode wettability, reduce free water activity, and suppress HER. These effects collectively establish ordered Zn^2+^ transport pathways, optimize electric field distribution, and promote uniform deposition [76]. The proposed mechanism directly translates to modified deposition kinetics and superior morphology. Chronoamperometry tests (Fig. 3h) show that with AM, Zn deposition rapidly transitions to a stable 3D growth mode, whereas the AM-free electrolyte exhibits prolonged, uneven 2D diffusion—consistent with guided nucleation and uniform flux. Among the AM-containing electrolytes, distinct kinetic behaviors are observed: 0.1 M exhibits a delayed stabilization with a pronounced current dip, reflecting nucleation-limited kinetics from incomplete HNI formation, while 0.5 M shows a slightly lower stable current with gradual drift, indicating transport limitations from excessive HNI thickness. Only 0.3 M achieves rapid stabilization with the highest stable current, demonstrating the optimal balance between sufficient nucleation sites and unimpeded ion transport. This controlled process yields dramatically improved electrode morphology.

Contact angle measurements (Fig. 3i) demonstrate excellent and sustained wettability in the AM system (angle as low as 11° after cycling), which promotes uniform ion transport. Consequently, SEM images reveal that AM leads to remarkably smooth and densely stacked Zn layers, in stark contrast to the dendritic and loose structure formed without AM. The consistency of this nucleation process and the resulting superior morphology are maintained throughout long-term cycling. Scanning electron microscopy (SEM) images of Zn anodes after 300 h of cycling at 1 mA cm^−2^ and 1 mAh cm^−2^ confirm the sustained formation of smooth and compact surfaces in the AM-modified electrolyte, in stark contrast to the inhomogeneous deposits formed without AM (Fig. S15). Remarkably, this uniform interfacial control is robust even under more demanding conditions. SEM images obtained after cycling at higher current densities and capacities (5 mA cm^−2^, 2.5 mAh cm^−2^ and 10 mA cm^−2^, 10 mAh cm^−2^) consistently show that the HNI effectively suppresses dendrites and cracks, whereas severe degradation occurs in the bare electrolyte (Figs. S16 and S17).

### Morphological Evolution and Structural Characterization of the HNI

**Fig. 4:**
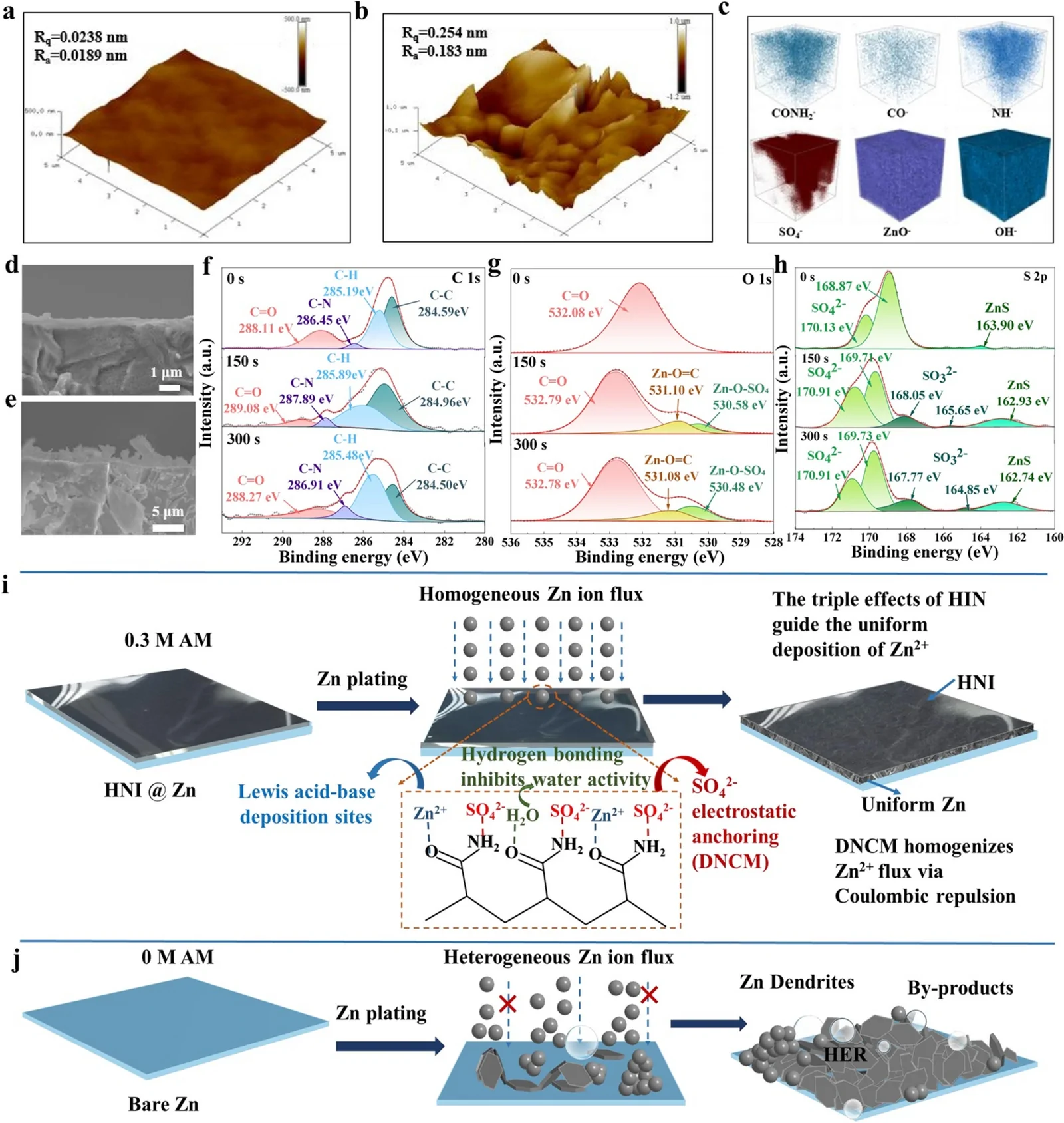
Structural and Chemical State Characterization of HNI. AFM images of Zn anode surfaces in Zn//Zn symmetric cells after 100 h cycling at 1 mA cm^−2^ and 1 mAh cm^−2^ in **a** 0 M AM electrolyte and **b** 0.3 M AM electrolyte. **c** Three-dimensional rendering of TOF–SIMS intensity under 4500 s sputtering in negative ion mode on the cycled Zn anode surface. Cross-sectional SEM images of Zn anodes from Zn//Zn symmetric cells after 100 h of cycling at 1 mA cm^−2^ and 1 mAh cm^−2^ in **d** 0 M AM electrolyte and **e** 0.3 M AM electrolyte. XPS fitting peaks of **f** C 1*s*, **g** N 1*s,* and **h** S 2*p* at different sputtering depths on the zinc anode surface after the Zn//Zn symmetric cell cycled for 100 h at 1 mA cm^−2^ and 1 mAh cm^−2^ in 0.3 M AM electrolyte. **i** HNI-mediated regulation of Zn^2+^ flux homogenization and uniform deposition. **j** Zn^2+^ deposition behavior of bare Zn anode in 0 M AM electrolyte

The uniform interfacial control enabled by the HNI, as evidenced by this suite of macroscopic to microscopic morphologies across varied conditions, is further quantified and corroborated at the nanoscale and through direct chemical analysis. The uniform interfacial control enabled by the HNI, as evidenced by the macroscopic morphology in Fig. 3i, is further corroborated at the nanoscale and through direct chemical analysis. Atomic force microscopy (AFM) provides quantitative confirmation of the pronounced smoothing effect. In the absence of AM, the Zn anode exhibits a rough surface (R_q_ = 0.254 nm, R_a_ = 0.183 nm, Fig. 4a), indicative of uneven nucleation. In contrast, with 0.3 M AM, the surface becomes remarkably smooth, with roughness values reduced by an order of magnitude (R_q_ = 0.0238 nm, R_a_ = 0.0189 nm, Fig. 4b). Cross-sectional scanning electron microscopy (SEM) visually confirms this distinct interfacial transformation. A dense and continuous film forms on the Zn anode cycled in the AM-containing electrolyte (Fig. 4d), in sharp contrast to the porous, dendritic structure obtained without AM (Fig. 4e). This compact and flat morphology remains consistent across various AM concentrations (Figs. S18 and S19). At 0.1 M AM, a thin but continuous HNI layer forms. At 0.3 M AM, the HNI reaches an optimal thickness (~ 5 μm), sufficient for effective protection without compromising interfacial kinetics. At 0.5 M AM, the HNI thickness further increases, but this excessive growth begins to hinder ion transport. This result directly demonstrating that an optimal thickness range exists for balancing protection and transport. Energy-dispersive X-ray spectroscopy (EDS) mapping further verifies that the film comprises C, N, O, S, and Zn elements (Fig. S20), providing direct evidence for the in situ formation of the HNI. This dramatic leveling effect directly visualizes the outcome of the flux homogenization mechanism proposed in Fig. 3g. The chemical basis and interfacial interactions within the HNI were unraveled by complementary depth-profiling techniques.

Time-of-flight secondary ion mass spectrometry (TOF–SIMS) 3D imaging (Figs. 4c and S21) provided spatial chemical mapping [77]. The uniform distribution of AM-derived fragments (C_3_H_5_NO_2_^−^, CONH_2_^−^, and CO^−^) across the analyzed depth confirms the structural integrity of the HNI in the bulk phase. Concurrently, the widespread signals of ZnO^−^ and OH^−^ reflect Zn^2+^ coordination (Zn–O = C) and a pervasive hydrogen-bonding network. Crucially, the tri-modal distribution and co-localization of NH^−^, SO_4_^2−^, and SO_3_^−^ signify sulfate enrichment at the interface via N–H···O = S hydrogen bonds, directly evidencing the formation of the DNCM. The co-localization of these species with H^+^, CH_2_^−^, and ZnS^−^ also hints at localized side reactions (e.g., SO_4_^2−^ reduction to ZnS), which the HNI framework confines and suppresses. X-ray photoelectron spectroscopy (XPS) depth profiling further deciphered the chemical states and bonding evolution (Fig. S22a). The survey spectrum confirmed the presence of C, O, N, S, and Zn. In the C 1*s* spectrum (Fig. 4f), characteristic peaks at 288.11 eV (C = O) and 286.45 eV (C-N) verified AM accumulation. With sputtering, the C = O peak shifted to 288.27 eV, while the corresponding O 1*s* peak (Fig. 4g) shifted upward to 532.78 eV. After 150 s, a new O 1*s* peak emerged at 531.10 eV, assigned to Zn–O = C coordination, accompanied by a negative shift of the Zn 2*p*_3/2_ peak to 1022.4 eV (Fig. S22b). These changes provide direct evidence for the strong carbonyl-Zn^2+^ coordination that establishes fixed nucleation sites. In the S 2*p* spectra (Fig. 4h), the positive shifts of S 2*p*_3/2_ (to 170.91 eV) and S 2*p*_1/2_ (to 169.73 eV) indicate SO_4_^2−^ participation via Zn–O-SO_4_ bonding. The concomitant appearance of an SO_3_^2−^ peak at 168.05 eV (shifting to 167.77 eV) and a negative shift of the N–H peak (Δ = -0.16 to 399.18 eV in Fig. S22c) confirm the N–H···O = S hydrogen bonding within the interface. Additional peaks suggest Zn-N interaction (396.73/399.09 eV).

These findings validate the in situ formation of a graded HNI. Its multi-tiered structure integrates the key interactions—C = O–Zn^2+^ coordination, SO_4_^2−^ anchoring, and a dense hydrogen-bond network—which work in concert to enable the triple synergistic mechanism illustrated in Fig. 4i: guided nucleation, flux homogenization, and interfacial stabilization. In stark contrast, the absence of this protective interphase leads to the scenario depicted in Fig. 4j: uncontrolled dendrite growth exacerbated by severe side reactions, ultimately precipitating rapid cell failure.

### HNI Modification Enables Ultra-Long Cycling Stability of Zn Anode

The formation and function of the HNI depend critically on achieving an optimal AM concentration, which must balance sufficient interfacial monomer supply against detrimental bulk effects. To identify this optimum, we screened Zn//Zn symmetric cell cycling across a wide concentration range (0.01–0.9 M) under demanding conditions (10 mA cm^−2^, 5 mAh cm^−2^). The results (Fig. S23) showed that cycling stability first improved, peaked at 0.3 M, and then declined. This trend is attributed to two competing factors: At low concentrations (e.g., < 0.1 M), interfacial AM coverage is incomplete, resulting in inadequate protection; at high concentrations (≥ 0.5 M), the increased monomer content elevates solution viscosity and raises the thermodynamic propensity for spontaneous polymerization, which can hinder ion transport and interfacial dynamics. Accordingly, 0.1, 0.3, and 0.5 M were selected as representative points below, at, and above the optimal window for subsequent study, with 0.3 M as the primary focus.

**Fig. 5:**
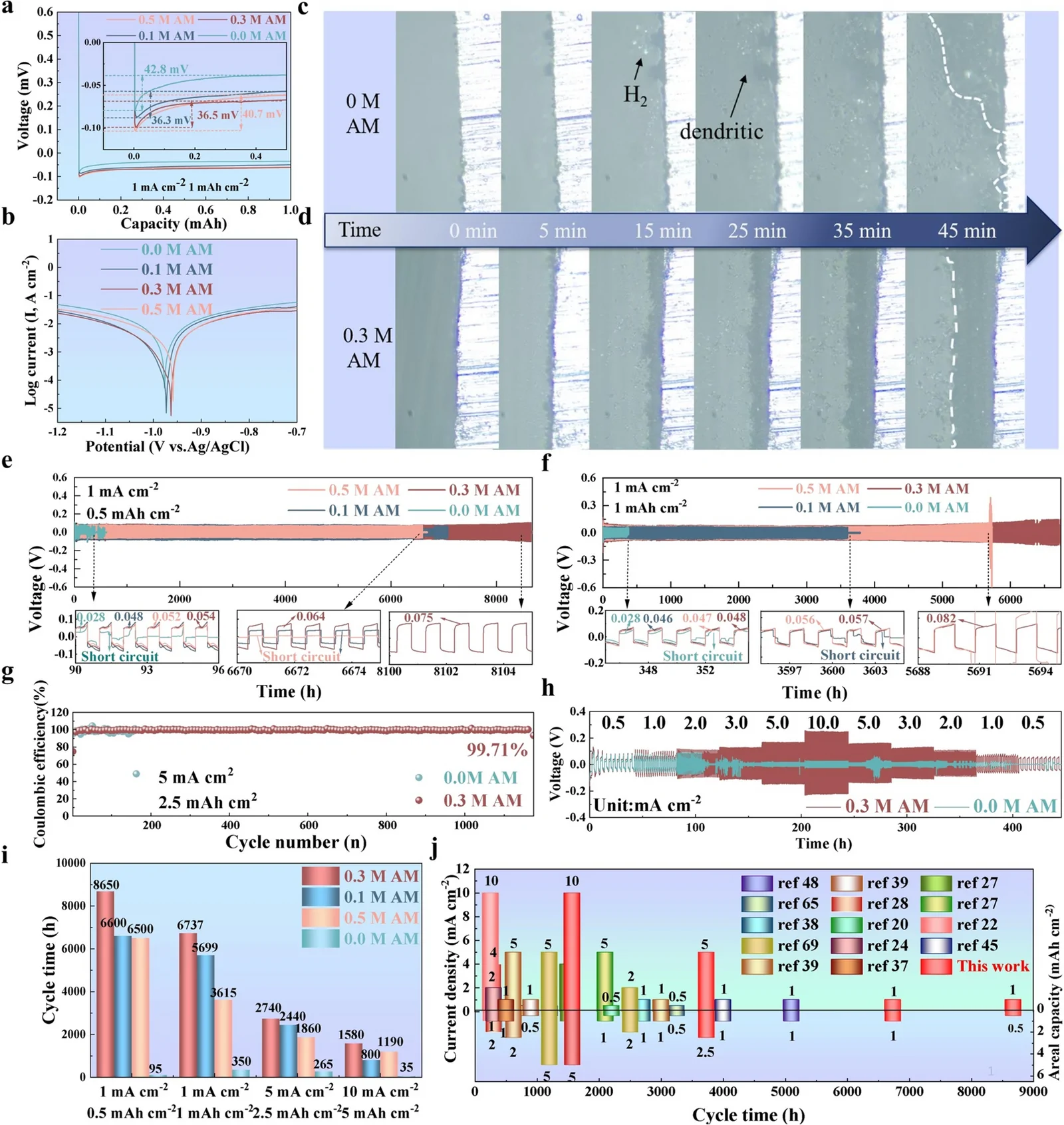
High-Performance Zn Deposition Guided by HNI. **a** Nucleation overpotential of electrolytes with different concentrations. **b** Tafel plots of Zn//Ti asymmetric cells in different electrolytes; in situ optical microscopy images of Zn^2+^ deposition process on Zn foil at 5 mA cm^−2^ in **c** 0.0 M AM electrolyte and **d** 0.3 M AM electrolyte. Cycling performance of Zn//Zn symmetric batteries under different current densities and areal capacity densities in electrolytes with varying concentrations of AM: **e** 1 mA cm^−2^, 0.5 mAh cm^−2^ and **f** 1 mA cm^−2^, 1 mAh cm^−2^. **g** Rate performance of Zn//Zn symmetric cells with 0.0 M AM and 0.3 M AM electrolytes. **h** Coulombic efficiency of Zn//Cu asymmetric cells at 5 mAh cm^−2^ in 0.0 M AM and 0.3 M AM electrolytes.** i** Comparison of cycling performance between Zn//Zn symmetric batteries in electrolytes with various AM concentrations at different current densities and areal capacity densities. **j** Comparison of cycling lifespan in this work with published literature data

The synergistic interfacial regulation and stabilized morphology ultimately translate into superior electrochemical performance. The introduction of AM leads to the in situ formation of a HNI that fundamentally optimizes the interfacial electrochemistry for zinc deposition. The nucleation overpotential, a key metric for uniformity, decreased from 42.8 mV in the 0 M AM electrolyte to 36.3 and 36.5 mV in 0.1 and 0.3 M AM electrolytes, respectively (Fig. 5a), indicating a lowered energy barrier for nucleation. Cyclic voltammetry of Zn//Ti cells further confirmed highly reversible plating/stripping with more favorable reaction kinetics in AM-containing electrolytes (Fig. S24). Tafel plots (Fig. 5b) reveal that all AM-modified electrolytes exhibit more positive corrosion potentials and lower corrosion current densities than the bare system, confirming that HNI formation effectively suppresses corrosive side reactions. Notably, the 0.3 M AM electrolyte shows the most negative value on the logarithmic current axis, corresponding to the lowest absolute corrosion current density and thus the best corrosion resistance. This concentration-dependent trend aligns perfectly with the optimized interfacial stability: 0.3 M AM achieves the optimal balance, while 0.1 M shows moderate improvement and 0.5 M exhibits slight decline due to transport limitations, further demonstrating that 0.3 M is the optimal concentration for maximizing protection, underscoring the HNI's role in suppressing corrosive side reactions. This optimized interface fundamentally redirects Zn deposition from chaotic to uniform. In situ optical microscopy captured the stark contrast in real-time: Within 45 min at 5 mA cm^−2^, the 0 M AM electrolyte fostered large, loose dendrites from localized nuclei (Fig. 5c), whereas the 0.3 M AM system facilitated smooth and homogeneous plating from the outset (Fig. 5d). Post-mortem analysis via ultra-depth microscopy (Fig. S25) and confocal laser scanning microscopy (CLSM, Fig. S26) solidified this observation, showing a rough, dendritic surface for the former and an exceptionally smooth one for the latter. These multi-scale visualizations confirm the HNI’s spatial guidance over Zn^2+^ plating. The HNI achieves this guidance without compromising ionic transport. Electrochemical impedance spectroscopy (EIS) showed that the charge transfer resistance remained low (Fig. S27a), confirming unimpeded ion conduction. Furthermore, post-cycling XRD analysis indicated that AM addition effectively retarded the formation of detrimental by-products (Fig. S27b), highlighting the interphase’s role in mitigating parasitic reactions.

The synergistic effects of the HNI translate into exceptional full-cell performance. Zn//Zn symmetric cells with the 0.3 M AM electrolyte achieved ultra-stable cycling for nearly 8650 h at 1 mA cm^−2^/0.5 mAh cm^−2^ (Fig. 5e) and over 6700 h at 1 mA cm^−2^/1 mAh cm^−2^ (Fig. 5f), outperforming the AM-free system by approximately 90 and 20 times, respectively. The authenticity of this representative 8650h cycling data is directly evidenced by the original test system screenshot (Fig. S28). This stability extended to high rates, with stable operation for ~ 2740 h at 5 mA cm^−2^ (Fig. S29) and ~ 1600 h at 10 mA cm^−2^ (Fig. S30). Critically, this order-of-magnitude enhancement in cycling life was rigorously reproducible. Independent replicate tests across key conditions—including 1 mA cm^−2^ (0.5 and 1 mAh cm^−2^) and 5 mA cm^−2^—consistently demonstrated that cells forming the HNI reliably achieved thousands of hours of stable operation, in stark contrast to the rapid failure of control cells (Fig. S31). Rate performance tests further confirmed the kinetic adaptability of the hydrated network interphase (Fig. 5g). The 0.3 M AM cell remained stable across stepwise current changes (0.5 → 10 → 0.5 mA cm^−2^) and fully recovered its initial voltage profile, demonstrating excellent reversibility. By contrast, the AM-free electrolyte failed at 2.0 mA cm^−2^ (Fig. S32). These results collectively highlight the critical role of hydrated network interphase in enabling highly reversible and dendrite-free Zn deposition under varied operating conditions. Zn//Cu asymmetric cells further demonstrate the enhanced reversibility. At 1 mA cm^−2^, the cycling life and Coulombic efficiency (CE) improved substantially with AM addition: from only ~ 200 cycles (98.74% CE) without AM to 2000 cycles (99.68% CE) with 0.1 M AM and 1600 cycles (99.66% CE) with 0.3 M AM (Fig. S33). At a higher current of 5 mA cm^−2^, the 0.3 M AM system maintained a high CE of 99.71% for 1150 cycles, starkly contrasting the early short-circuiting (~ 150 cycles) of the AM-free cell (Fig. 5h). A comprehensive comparison across all tested conditions consistently underscores the superior stability of the HNI-based anodes (Fig. 5i). Ultimately, when benchmarked against other recently reported advanced systems, the cycling longevity achieved by this strategy is highly competitive (Fig. 5j), which conclusively validates the HNI as an efficient interfacial design for achieving durable and dendrite-free zinc metal anodes.

### High-Performance Zn//I_2_ Full Cell Enabled by the HNI

**Fig. 6:**
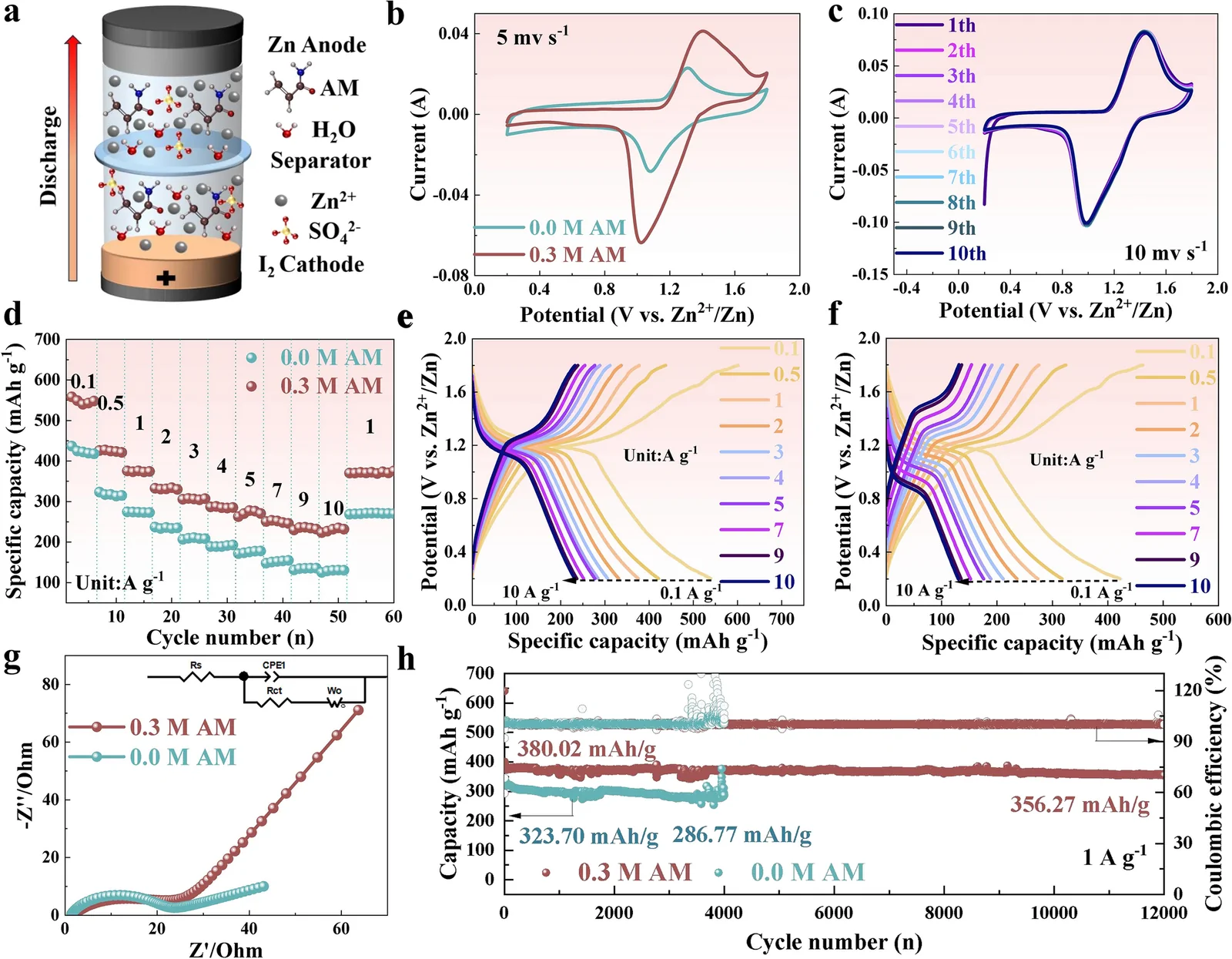
High-Performance Zn-I₂ Full Cell Enabled by the HNI. **a** Structure of the Zn//I_2_ full cell. **b** CV curves of the Zn//I_2_ full cell in 0 M AM and 0.3 M AM electrolytes (scan rate of 10 mV s^−1^). **c** CV curves of the Zn//I_2_ full cell in 0.3 M AM electrolyte at different cycle numbers (scan rate of 10 mV s^−1^). **d** Rate performance of the Zn//I_2_ full cell in 0 M AM and 0.3 M AM electrolytes. GCD curves of the Zn//I_2_ full cell in **e** 0.3 M AM electrolyte and **f** 0 M AM electrolyte. **g** EIS plots and **h** long-cycle life curves with Coulombic efficiency of the Zn//I_2_ full cell in 0 M AM and 0.3 M AM electrolytes

Iodine offers not only high  theoretical capacity and output voltage (422 mAh g^−1^ at 1.83 V vs. Zn^2+^/Zn, based on the I^−^/I_2_ /I^+^ redox reaction) [78, 79] but also ecological advantages, including environmental friendliness and non-toxicity. Therefore, in this chapter, I_2_ was used as the cathode material, Zn foil as the anode material, and 0.3 M AM and 0 M AM as electrolytes to assemble a complete aqueous zinc-ion battery for testing its stability and cycling performance, as shown in Fig. 6a. First, the CV curves of Zn//I_2_ full cells assembled with 0.3 M AM and 0 M AM electrolytes were tested. The CV diagrams reveal  redox process of iodine species within the voltage window of 1.2-1.6 V. The single pair of redox peaks  may arise from the overlapping electrochemical responses of the sequential I^−^/I_2_ and I_2_/I^+^ conversions, as the potential windows are in close proximitly. At both 5 mV s^−1^ (Fig. S34a) and 10 mV s^−1^ (Fig. 6b) scan rates, the redox peak area of the 0.3 M AM electrolyte is significantly larger than that of 0 M AM, demonstrating higher redox currents in the cell with 0.3 M AM electrolyte during the process. This suggests faster ion transport kinetics in the 0.3 M AM system. Furthermore, to evaluate the structural stability of the Zn//I_2_ battery with 0.3 M AM electrolyte, CV curves were measured over 10 cycles at scan rates of 5 mV s^−1^ (Fig. S34b) and 10 mV s^−1^ (Fig. 6c). The CV curves from the 2nd to the 10th cycle show nearly complete overlap after the initial cycle, further demonstrating that the Zn//I_2_ battery in 0.3 M AM electrolyte not only exhibits rapid ion transport kinetics but also maintains a stable electrochemical activity window.

To more intuitively characterize its stability, the rate performance of encapsulated Zn//I_2_ full batteries in two different electrolytes was further tested, as shown in Fig. 6d. The rate performance analysis reveals that after adding AM, the specific capacities at various current densities are consistently higher than those of the electrolyte without AM. This indicates that the Zn//I_2_ reaction becomes more complete with AM-containing electrolyte, achieving fuller utilization of the I_2_ cathode. The GCD curves (Fig. 6e) demonstrate that the Zn//I_2_ battery with 0.3 M AM electrolyte exhibits stable charge/discharge platforms across current densities ranging from 0.1 to 10 A g^−1^. Specifically, it delivers specific capacities of 539.89 mAh g^−1^ at 0.1 A g^−1^, 373.57 mAh g^−1^ at 1 A g^−1^ and maintains 231.75 mAh g^−1^ even at 10 A g^−1^. When the current density returns to 1 A g^−1^, the capacity remains at 372.33 mAh g^−1^. In contrast, the Zn//I_2_ battery with 0 M AM electrolyte (Fig. 6f) shows unstable charge/discharge platforms over the same current density range, with its capacity decreasing from 418 mAh g^−1^ at 0.1 A g^−1^ to 130 mAh g^−1^ at 10 A g^−1^—nearly half that of the AM-containing system. These results indicate that AM primarily functions at the zinc anode interface, where it forms a hydrated network interphase without affecting the iodine cathode. This interphase significantly suppresses side reactions (e.g., HER and corrosion) and enhances ion transport kinetics. EIS measurements (Fig. 6g) corroborate this, showing lower charge transfer resistance (Rct = 18.5 Ω) and a steeper Warburg slope in the 0.3 M AM cell, confirming improved conductivity and diffusion. Long-term cycling (Fig. 6h) further highlights these benefits: The 0.3 M AM cell retained 356.27 mAh g^−1^ (89.15% capacity) after 12,000 cycles at 1 A g^−1^, whereas the AM-free cell failed after ~ 4000 cycles. Overall, the AM-induced interphase enables uniform Zn deposition, mitigates anode degradation, and significantly boosts full-cell performance and longevity.

## Conclusion

This work confirms that optimizing the electrolyte of aqueous zinc-ion batteries to form a HNI can significantly reconstruct the solvation structure at the electrode interface, thereby achieving unprecedented long-term cycling stability. The AM additive spontaneously adsorbs and polymerizes on the surface of the zinc anode, constructing a multifunctional protective network with a threefold synergistic mechanism: 1) Lewis acid–base coordination (Zn^2+^···O = C) provides preferential nucleation sites to guide directional Zn^2+^ deposition and suppress erratic migration; 2) hydrogen bonding (N–H···O = S) and electrostatic interactions form DNCM, alleviating concentration polarization and guiding uniform Zn^2+^ deposition; and 3) C = O···H–O-H hydrogen bonding immobilizes water molecules, reducing free water activity and thereby suppressing hydrogen evolution. The 0.3 M AM electrolyte delivers excellent performance: Zn//Zn cells cycle stably for 8650 h (1 mA cm^−2^/0.5 mAh cm^−2^), 6600h (1 mA cm^−2^/1 mAh cm^−2^), and 1600 h (10 mA cm^−2^/5 mAh cm^−2^); Zn//Ti cells reach 99.71% Coulombic efficiency (5 mA cm^−2^); and full cells retain 356.27 mAh g^−1^ (89.15% retention) after 12,000 cycles (1 A g^−1^). This interfacial engineering strategy provides a transformative way to develop high-performance Zn anodes for aqueous batteries.

## Supplementary Information

Below is the link to the electronic supplementary material.

Supplementary file1 (DOCX 14061 KB)
