# Supplementary material for: Hydrated Network Interphase with Dynamic Negatively Charged Microregion Enables Ultra-Stable Aqueous Zinc-Ion Batteries
Source: Nanomicro Lett. 2026 Jun 29;18:418. doi: 10.1007/s40820-026-02262-0 (PMC13315086; doi:10.1007/s40820-026-02262-0)
Supplement: Supplementary file 1 — Supplementary file1 (DOCX 14061 KB) [file 40820_2026_2262_MOESM1_ESM.docx]

Supporting Information for

**Hydrated Network Interphase with Dynamic Negatively Charged Microregion Enables Ultra-Stable Aqueous Zinc-Ion Batteries**

Yin Yang^1,#^, Xiaofang Wang^1, #^, Xin Chen^1^, Jia Yao^1^, Daigan Wang^1^, Luyang Ge^1^, Fei Wang^1^, Lin Lv^1^, Li Tao^1^, Hao Wang^1, *^, Houzhao Wan^1,*^

^1^ Hubei Key Laboratory of Micro-Nanoelectronic Materials and Devices, School of Integrated Circuits, Hubei University, Wuhan 430062 P. R. China

^#^ Yin Yang and Xiaofang Wang contributed equally to this work.

* Corresponding authors. E-mail: [nanoguy@126.com](mailto:nanoguy@126.com) or [wangh@hubu.edu.cn](mailto:wangh@hubu.edu.cn) (Hao Wang); [houzhaow@hubu.edu.cn](mailto:houzhaow@hubu.edu.cn) (Houzhao Wan)

**S1 Methods**

**S1.1 Characterizations**

The XRD characterization in this study was performed using a Bruker D8 Advance X-ray diffractometer with a maximum radiation source power of 3 kW. Diffraction patterns were collected over the 2θ range of 5° to 90°. Microstructural observations were carried out with a JSM-7100F Schottky field emission scanning electron microscope (SEM) operating at an acceleration voltage of 15 kV, maintaining a working distance of 10 mm. Elemental composition and distribution analyses were conducted through energy-dispersive X-ray spectroscopy (EDS) coupled with the SEM system, covering elements from Be4 to Cf98. X-ray photoelectron spectroscopy (XPS) measurements were acquired using an ESCALAB 250Xi system (Thermo SCIENTIFIC) with an X-ray source operated at 15 kV and 10 mA. Raman spectroscopy was performed on a Renishaw spectrometer equipped with a 532 nm laser excitation source, with the laser power maintained at 50% of maximum output for both electrode materials and electrolyte analysis. Fourier-transform infrared (FT-IR) spectra were recorded in the range of 200-4000 cm^-1^ using a Thermo Fisher Scientific spectrometer.

Optical microscopy observations were conducted with a 10× objective lens (Shenzhen Aoswei Optical Instrument Co., Ltd.). Three-dimensional surface topography characterization was achieved using a 50× objective lens-equipped ultra-depth-of-field microscope (Chengdu Liyang Precision Electromechanical Co., Ltd.). Contact angle measurements were performed using a JY-82B Kruss DSA instrument (Germany). ^1^H NMR spectra were recorded on a Bruker Avance 400 (400 MHz) spectrometer, using D_2_O as solvent. HRMS was acquired from orbitrap LC/MS (Q Exactive) spectrometer.

**S1.2 Electrochemical Measurements**

Novel electrolytes were formulated by introducing varying concentrations of acrylamide (AM) as additives into conventional 2 M ZnSO_4_ electrolytes. The developed electrolytes were subsequently assembled into Zn//Zn symmetric cells and Zn//Cu half-cells for systematic electrochemical characterization using a Neware battery testing system. The testing protocol included evaluations of cycling stability and rate capability under specific current density conditions (0.5, 1, 2, 3, 5 and 10 mA cm^-2^) and areal capacity densities (0.5, 1 and 5 mAh cm^-2^). Coulombic efficiency measurements and polarization curve analyses were conducted with a charging cutoff voltage of 0.5 V. Comprehensive electrochemical characterizations including cyclic voltammetry (CV), linear sweep voltammetry (LSV), Tafel polarization, and electrochemical impedance spectroscopy (EIS) were performed using a CHI760E electrochemical workstation. For full-cell evaluations, Zn-I_2_ batteries underwent galvanostatic charge-discharge cycling tests (current density: 1 A g^-1^; voltage window: 0.2-1.8 V) and rate performance assessments on the Neware system. All experimental procedures were executed under controlled environmental conditions with laboratory temperature maintained at a constant 26°C to ensure measurement consistency.

**S1.3 Electrolyte conductivity test method**

Conductivity measurement by Ti//Zn half battery. This is done using electrochemical impedance spectroscopy (EIS) techniques with a frequency range of 100,000 Hz to 1 Hz. The ohmic resistance of the electrolyte takes the value of the low frequency end of the semicircle at the intersection of the x-axis of the Nyquist diagram. The final ion conductivity is calculated as follows:

$\sigma= \frac{L}{RS}$ (S1)

$L$ is the value of the membrane thickness，$S$ is the surface area of electrode，$R$ is the ohm resistance of the conductor.

**S1.4 Solid Zeta Potential**

Instrument Model: Anton Paar Surpass 3; Methodology: Based on the streaming potential method: A pressure gradient is applied parallel to the sliding surface, inducing directional migration of counterions in the diffuse layer under the pressure-driven flow, thereby generating a streaming current. Accumulated counterions on the low-pressure side create a reverse electric field, corresponding to a conduction current opposing the streaming current. When the forward (streaming) and reverse (conduction) currents reach equilibrium, the resulting electric potential under the applied pressure gradient is measured as the streaming potential.

**S2 Computational details**

**S2.1 Density functional theory method**

The DFT calculation was performed by the Vienna Ab-initio Simulation Package (VASP) [S1], and the exchange-correlation energy was approximately described by the Perdew-Burke-Ernzerhof (PBE) [S2] functional based on the generalized gradient approximation (GGA). In all calculation, a cut-off energy with a value of 450 eV [S3] was used for the plane wave basis, and the convergence criteria for the ionic relaxation and electronic self-consistent calculation were set to 0.02 eV Å-1 and 10-4 eV, respectively. The Zn (002) surface is modeled by a six-layers 5 × 5 surface supercell (150 Zn atoms of all), the Zn (101) surface is modeled by a ten-layers 2 × 4 surface supercell (80 Zn atoms of all) and the Zn (100) surface is moldeled by a ten-layers 4 × 3 surface supercell (120 Zn atoms of all). The long-range dispersion correction for the van der Waals interaction was implemented through the DFT-D3 method [S4, S5] in all calculation. The adsorption energy of the H_2_O or AM molecules on the Zn surfaces was computed from

$E_{-abs}=E_{-complexe}-E_{-Zn}-$ (S2)
where E _complexes is the energies of complexes after Zn surface adsorption by molecules, E __Zn_ is the energy of Zinc phases of different surfaces, and E _molecules is the energy of H_2_O or AM molecules. The Quantum chemical calculations were performed using the Gaussian16 program. The geometrical structures were optimized at the B3LYP/6-31+G (d) level and the weak interaction binding energys (Eb) were calculated at the B3LYP/6-311+G (d, p) level of theory. Based on the above results, the HOMO-LUMO and ESP of the solvents were analyzed by using Multiwfn and VMD software. In addition, the binding energys were attributed to electrostatic interactions (ion–dipole interaction) between the solvent and SO_4_^2-^ in electrolytes and was defined as follows:

$E_{b}=E_{x-y}-E_{x}-E_{y}$ (S3)
where Ex−y is the total energy of the SO_4_^2-^-solvent complexes and Ex and Ey are the energies of components x and y, respectively.

All quantum chemical calculations were performed by applying the density functional theory (DFT) method with the B3LYP level and 6-31+G (d, p) basis set using Gaussian package. The structural optimization of the molecules or ions in this work was determined by minimizing the energy without imposing molecular symmetry constraints. The transition state was determined by the QST2 method and was confirmed by the IRC calculations so that the reaction barriers can be obtained. Inaddition, the QUANTUM ESPRESSO was adopted to study the binding energies.

The calculations about ESP were carried out on Gaussian 09 package [S6]. Density functional theory (DFT) was used to optimize the structure, with B3LYP exchange-correlation functional and 6-311G was selected as calculation basis set [S7].

**Supplementary Figures**


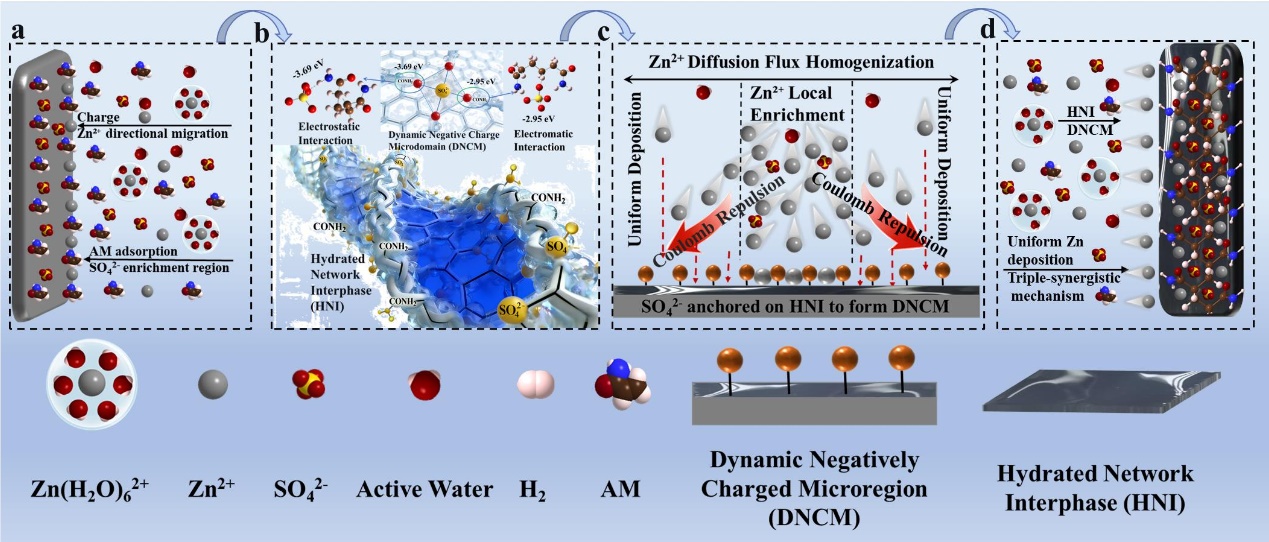


**Fig. S1 Schematic illustration of DNCM formation, dynamic maintenance, and function.** **a** Formation of HNI: Electrochemically enriched Zn^2+^ and SO_4_^2-^ triggers polymerization of pre-adsorbed AM, forming stable HNI layer. **b** Formation of DNCM: SO_4_^2-^ anchors within HNI via N-H···O=S bonding, creating negatively charged microregions. **c** Dynamic maintenance and function: Continuous ion migration dynamically maintains DNCM. Anchored SO_4_^2-^ homogenizes Zn^2+^ flux via Coulombic repulsion. **d** Uniform Zn^2+^ deposition: DNCM-mediated flux homogenization enables smooth, dendrite-free morphology


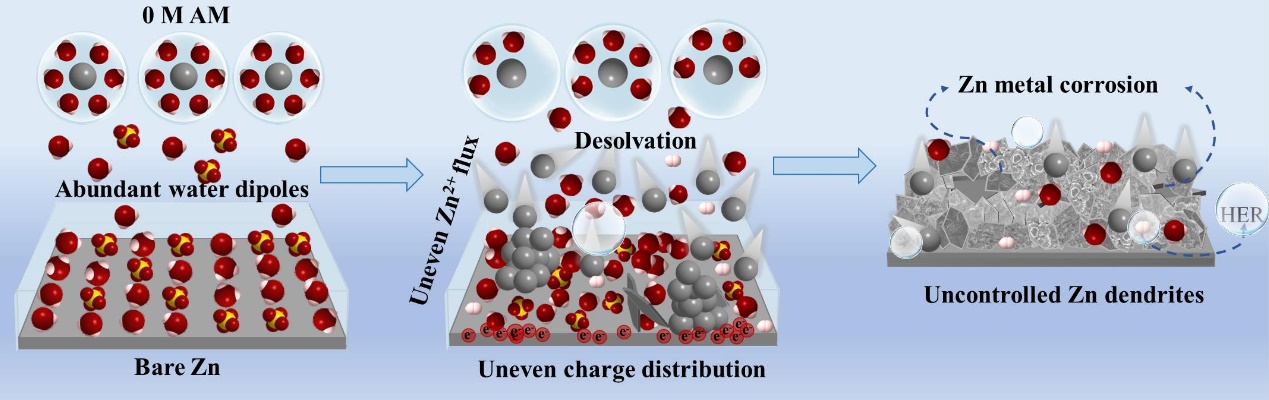


**Fig. S2** Schematic illustration of the Zn deposition process at the electrode interface in 0 M AM electrolyte (The Zn anode in AM-free ZnSO_4_ electrolyte exhibits severe dendrite growth, hydrogen evolution, and corrosion, leading to a drastically degraded cycling lifespan.)


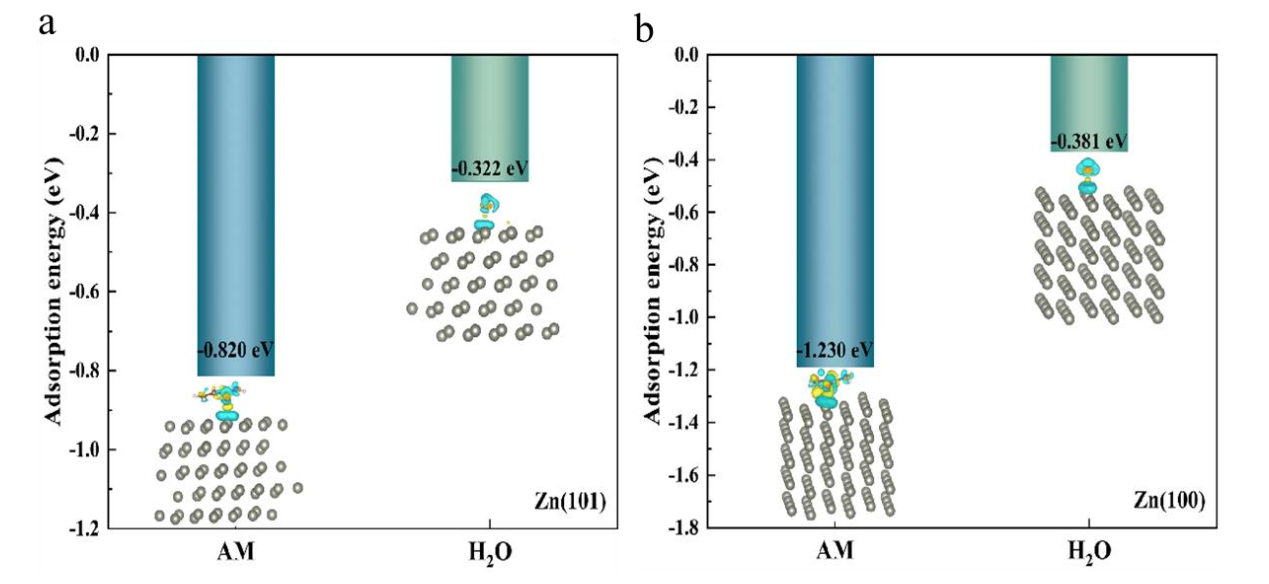


**Fig. S3** The adsorption energies of water molecules and AM molecules on the Zn surface are shown, with the corresponding adsorption models illustrated in the insets: **a** Zn (101) and **b** Zn (100)

**
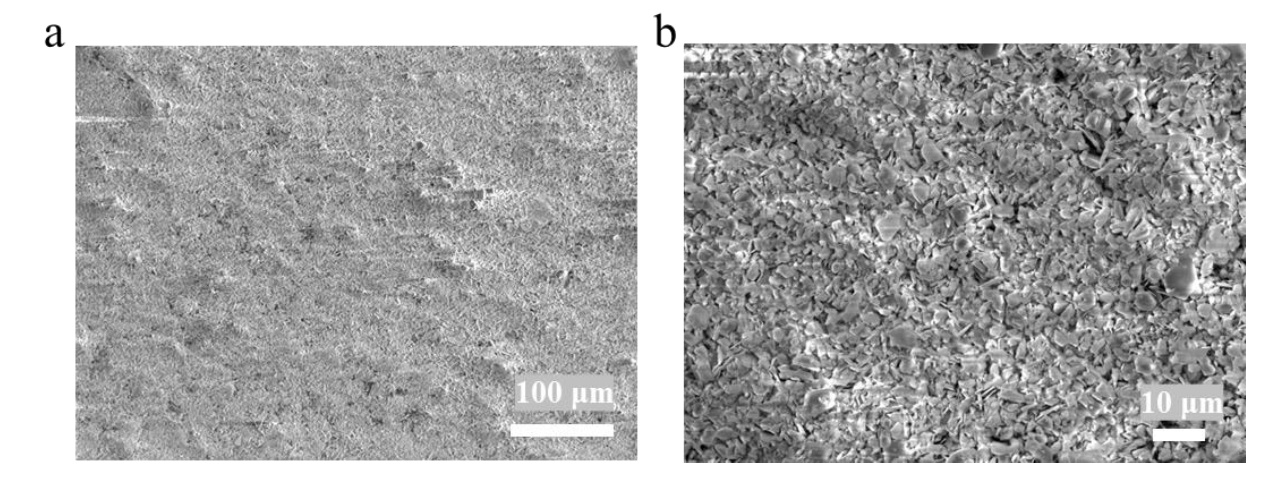
**

**Fig. S4** SEM images of Zn foil surfaces immersed in different solutions for 1 month: **a** Pure water solution. **b** AM aqueous solution

**
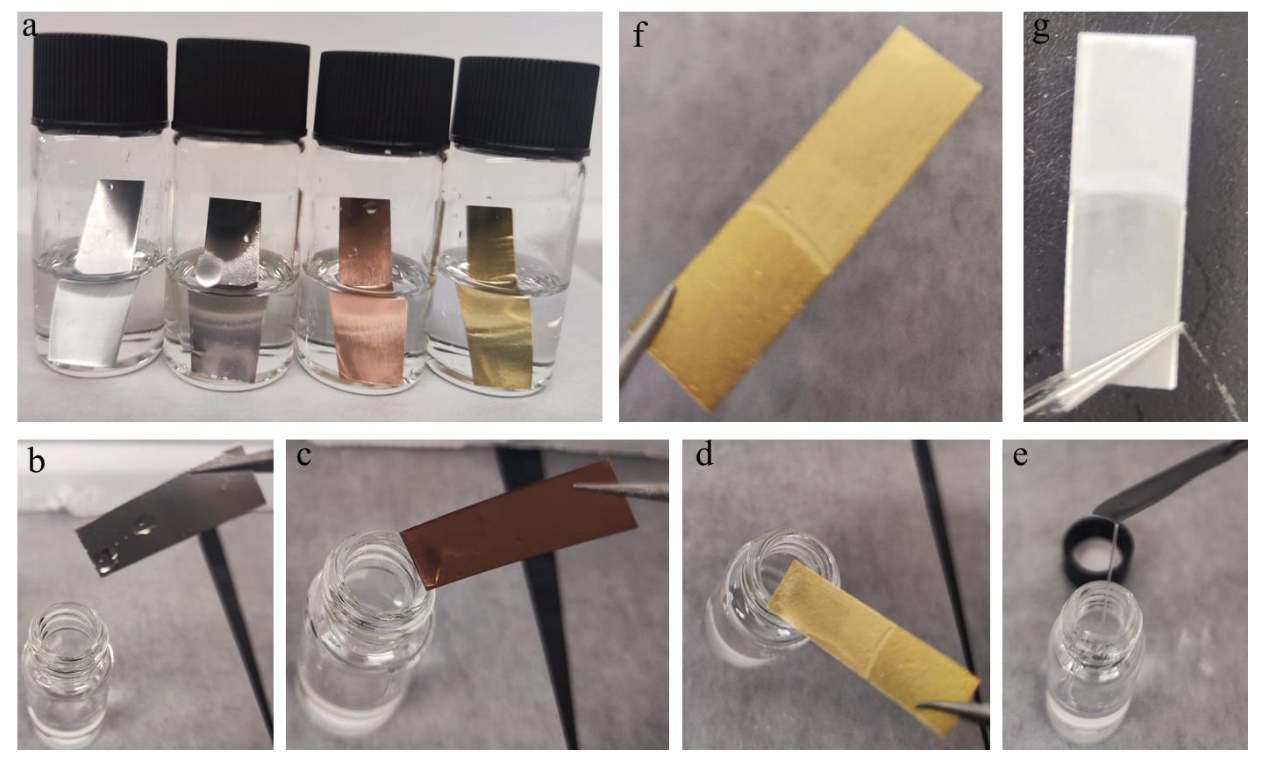
**

**Fig. S5 a** Digital photographs of various metal foils (from left to right: Ti foil, Zn foil, pure Cu foil, Zn-Cu alloy foil) immersed in 0.3 M AM electrolyte. **b** Ti foil after immersion. **c** Pure Cu foil after immersion. **d** Zn-Cu alloy foil after immersion. **e** Zn foil after immersion. **f** Magnified view of the Zn-Cu alloy foil (showing trace amounts of surface polymer induced by slight AM adsorption due to the Zn alloy composition). **g** Magnified view of the Zn foil (showing scant and non-uniform, unstable surface polymer)


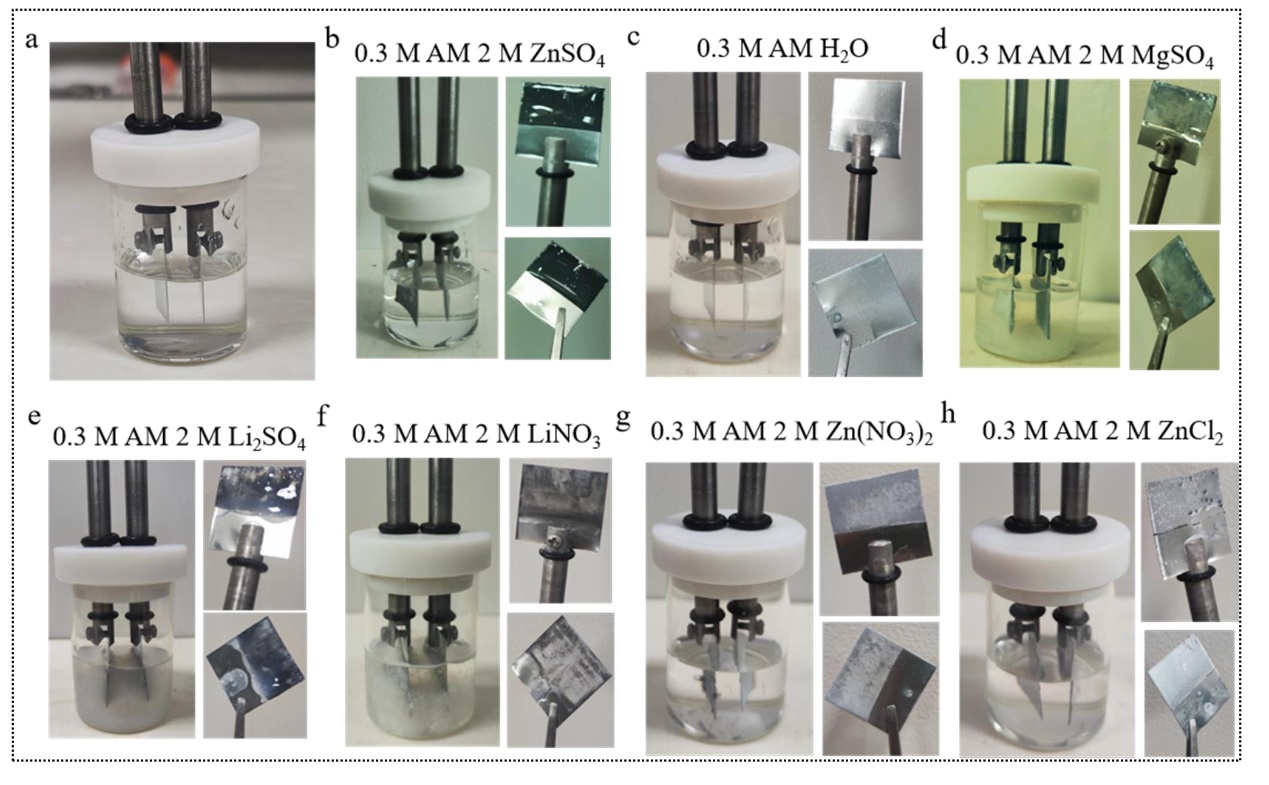


**Fig. S6 a** Experimental setup for Zn^2+^ plating/stripping cycling tests at 1 mA cm^-2^ in various electrolytes. Digital photographs of Zn electrodes after cycling in: **b** 0.3 M AM + 2 M ZnSO_4_ (Zn^2+^ + SO_4_^2-^) — uniform, transparent HNI observed. **c** 0.3 M AM + H_2_O (no Zn^2+^, no SO_4_^2-^) — no HNI formation. **d** 0.3 M AM + 2 M MgSO_4_ (SO_4_^2-^ only) and **e** 0.3 M AM + 2 M Li_2_SO_4_ (SO_4_^2-^ only) — white flocculent precipitates appear, but no structured HNI. **f** 0.3 M AM + 2 M LiNO_3_ (no Zn^2+^, no SO_4_^2-^) — no HNI. **g** 0.3 M AM + 2 M Zn(NO_3_)_2_ (Zn^2+^ only) and **h** 0.3 M AM + 2 M ZnCl_2_ (Zn^2+^ only) — no visible HNI. These results demonstrate that HNI formation requires the simultaneous presence of both Zn^2+^ and SO_4_^2-^

**Table S1** Summary of control experiments verifying the synergistic role of Zn^2+^ and SO_4_^2-^ in HNI formation

| Electrolyte | Zn^2+^ | SO_4_^2-^ | Observation after cycling | Interpretation | HNI |
| --- | --- | --- | --- | --- | --- |
| 0.3 M AM + 2 M ZnSO_4_ | √ | √ | Transparent, uniform, adherent HNI | Synergistic effect: Zn^2+^ initiates polymerization + SO_4_^2-^ directs assembly into network | √ |
| 0.3 M AM + 2 M Zn(NO_3_)_2_ | √ | × | No visible interphase | Zn^2+^ initiates polymerization but without SO_4_^2-^, chains cannot assemble into structured network | × |
| 0.3 M AM + 2 M ZnCl_2_ | √ | × | No visible interphase | Same as above | × |
| M AM + 2 M MgSO_4_ | × | √ | White flocculent precipitate (loose, non-adherent) | Electrochemically enriched SO_4_^2-^ induces salting-out of AM, but without Zn^2+^ catalysis, only physical aggregation occurs | × |
| 0.3 M AM + 2 M Li_2_SO_4_ | × | √ | White flocculent precipitate | Same as above | × |
| 0.3 M AM + 2 M LiNO_3_ | × | × | Minor precipitate | Possible weak Li^+^ effect or side reactions; no HNI formation | × |
| 0.3 M AM (aqueous) | × | × | No visible change | No trigger for polymerization or assembly | × |

**
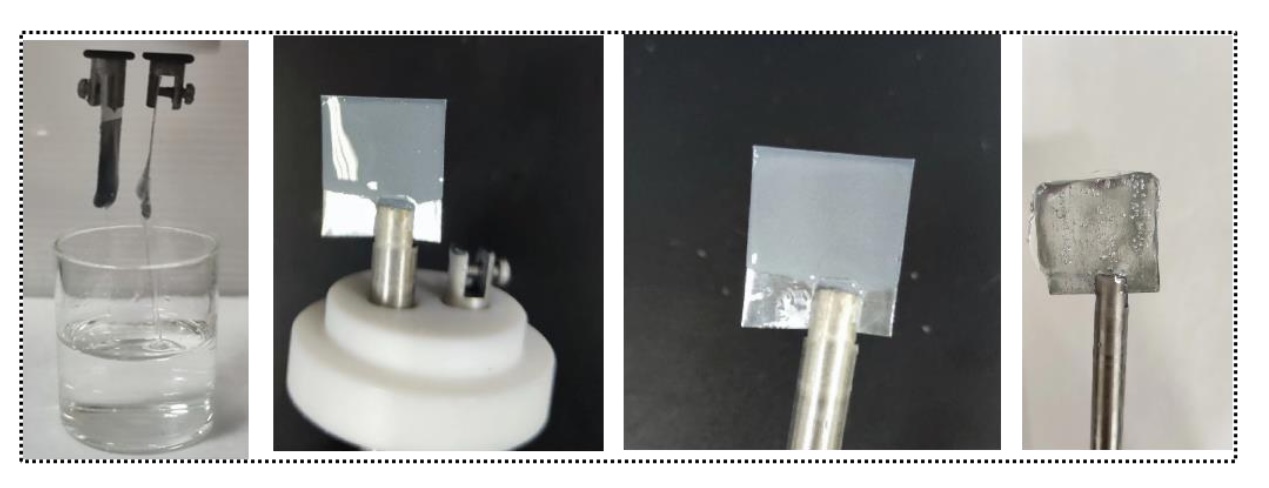
Fig. S7** Zn^2+^ is electroplated in an electrolyte consisting of 0.5 M AM and 2 M ZnSO_4_ at a current density of 10 mA cm^-2^


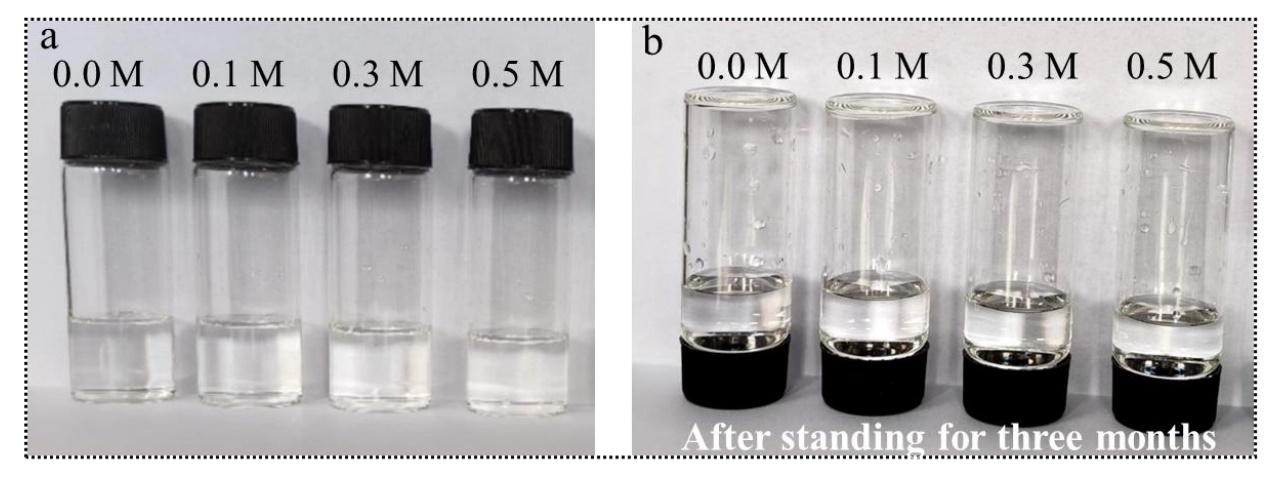


**Fig. S8** Electrolyte of 2 M ZnSO_4_ + x M AM (x = 0.0, 0.1, 0.3, 0.5): **a** AM fully dissolved and **b** State after 3 months


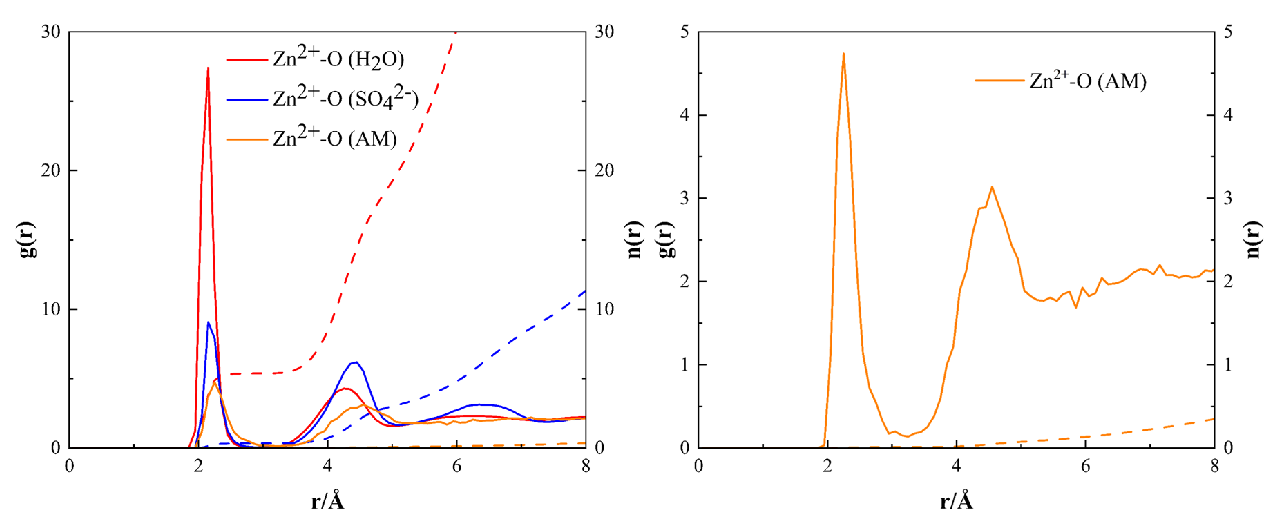


**Fig. S9** Zn^2+^-O Radial Distribution Functions for H_2_O, SO_4_^2-^ and AM Species in Aqueous Electrolyte

**
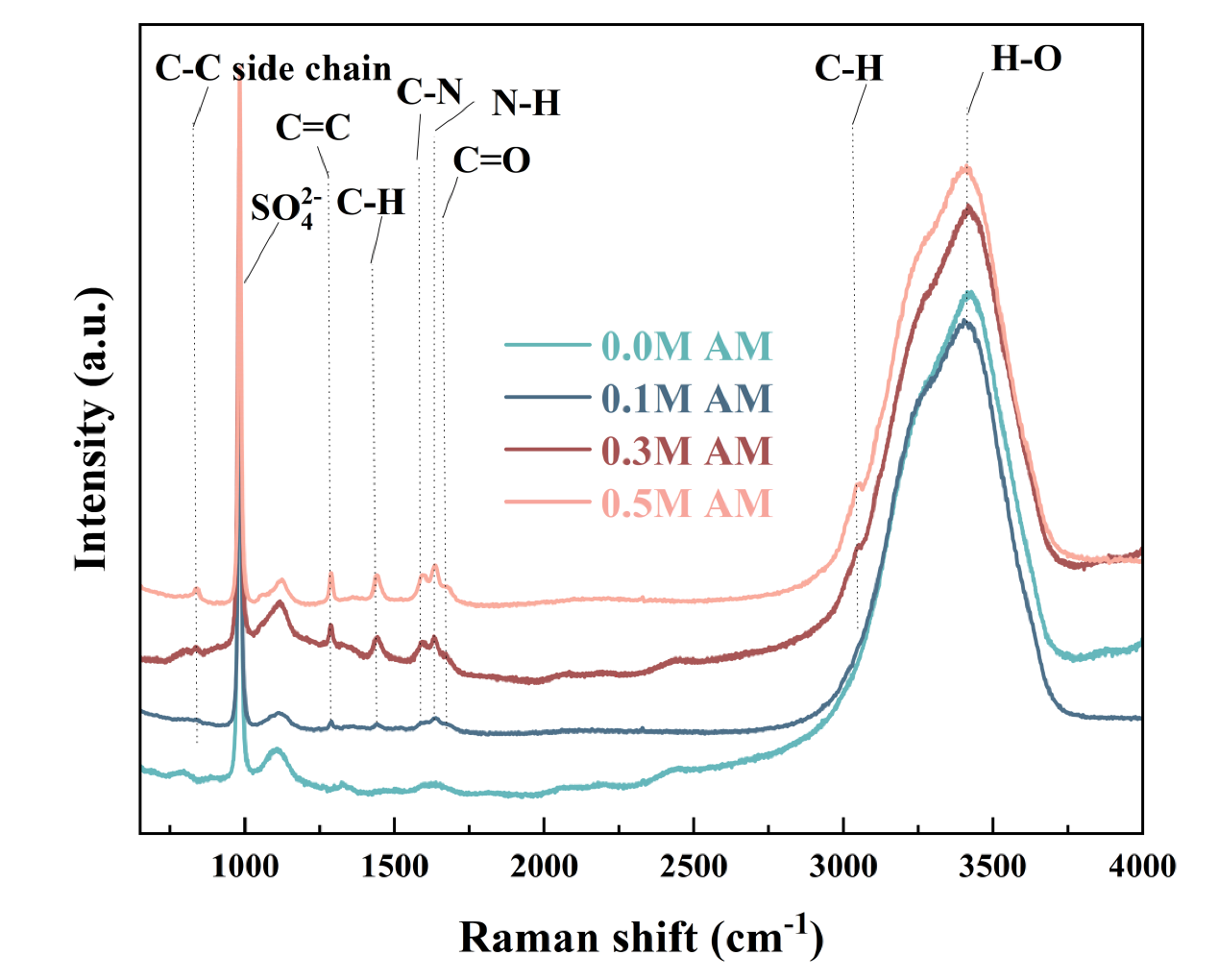
**

**Fig. S10** Raman spectroscopy of x M AM (x = 0.0, 0.1, 0.3, 0.5) electrolyte


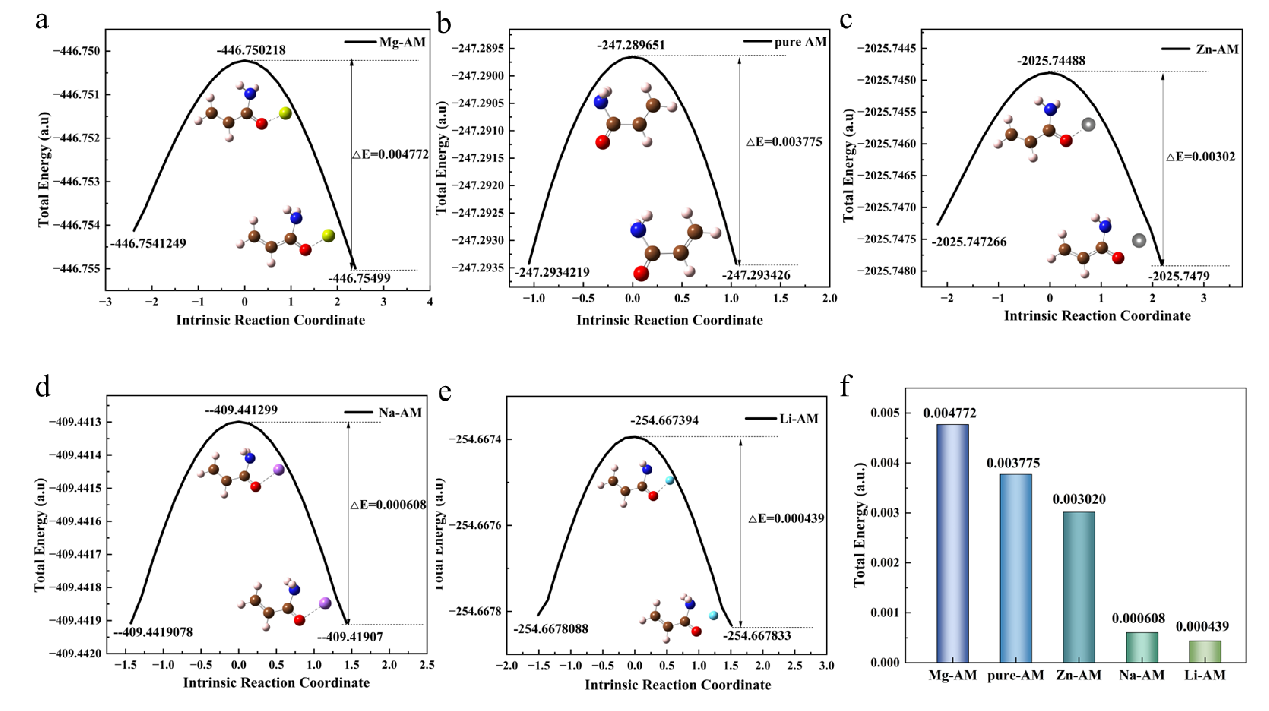


**Fig. S11** The free energy and reaction energy barriers of AM crosslinking reactions induced by various cations: **a** Mg^2+^ (hindering polymerization), **b** No cation added, **c** Zn^2+^ (Promote polymerization), **d** Na^+^ (Promote polymerization), **e** Li^+^ (Promote polymerization) and **f** Bar chart comparison of reaction energy barriers


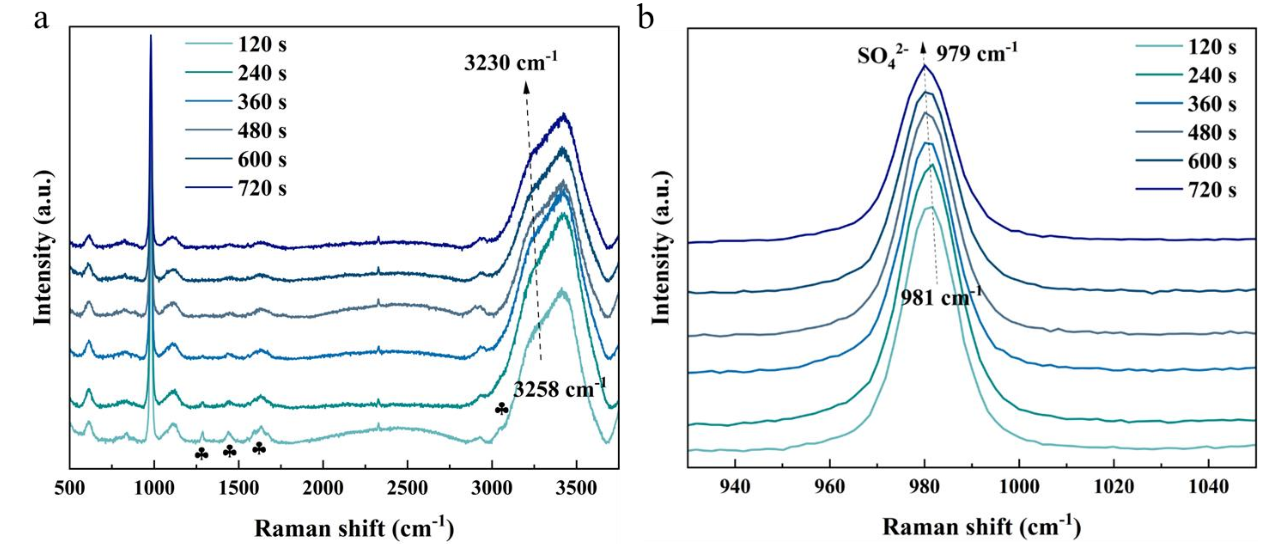


**Fig. S12 a** Time-dependent in situ Raman spectra of 0.3 M AM electrolyte during reaction. **b** Time-dependent in situ Raman spectra of SO_4_^2-^ signal peaks in 0.3 M AM electrolyte (The decrease in SO_4_^2-^ and H_2_O peak intensities indicated their incorporation into the interphase via electrostatic and hydrogen-bonding interactions with the amide N-H groups, also inducing slight redshift and regulating Zn^2+^ transport. The hydrophilicity of the interphase chains further restricted free water, weakening the H_2_O signal. A subtle redshift of the O-H peak (3258 → 3230 cm^-1^) corroborated the reduced interfacial free water content)


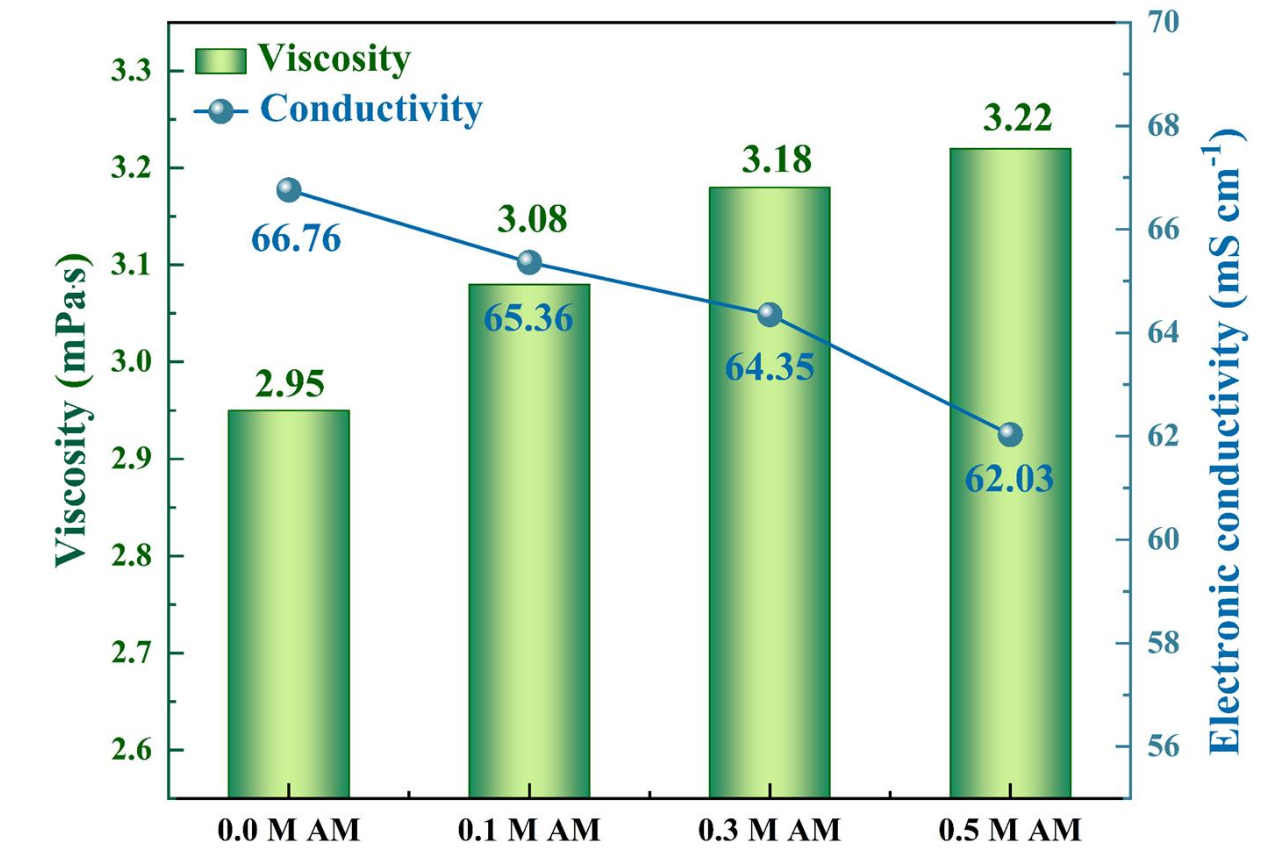


**Fig. S13** Viscosity and ionic conductivity of electrolytes with different concentrations of AM

**
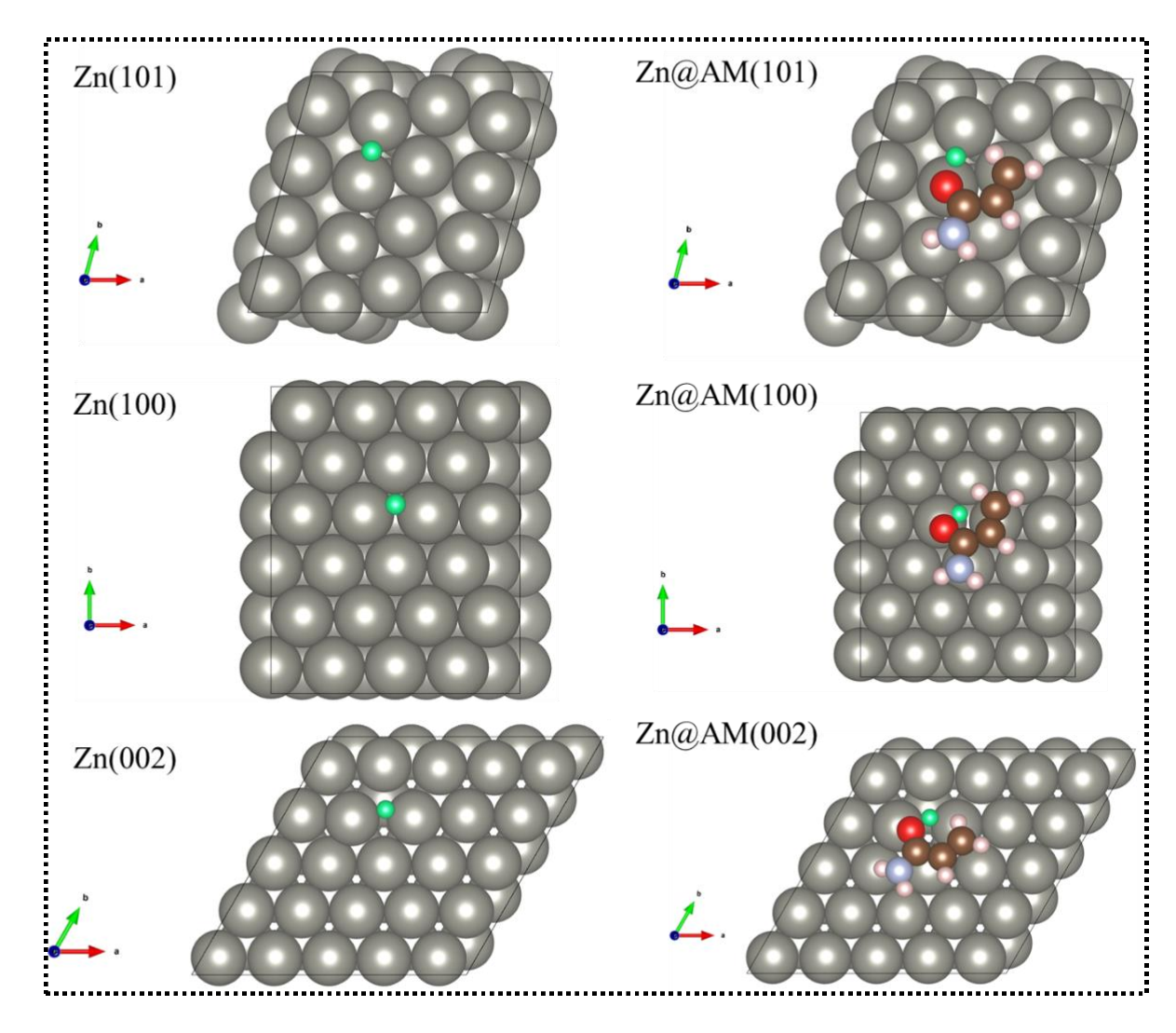
Fig. S14** The HER energy barrier reaction model on the surface of bare Zn (left) and Zn anode with AM adsorption (right)


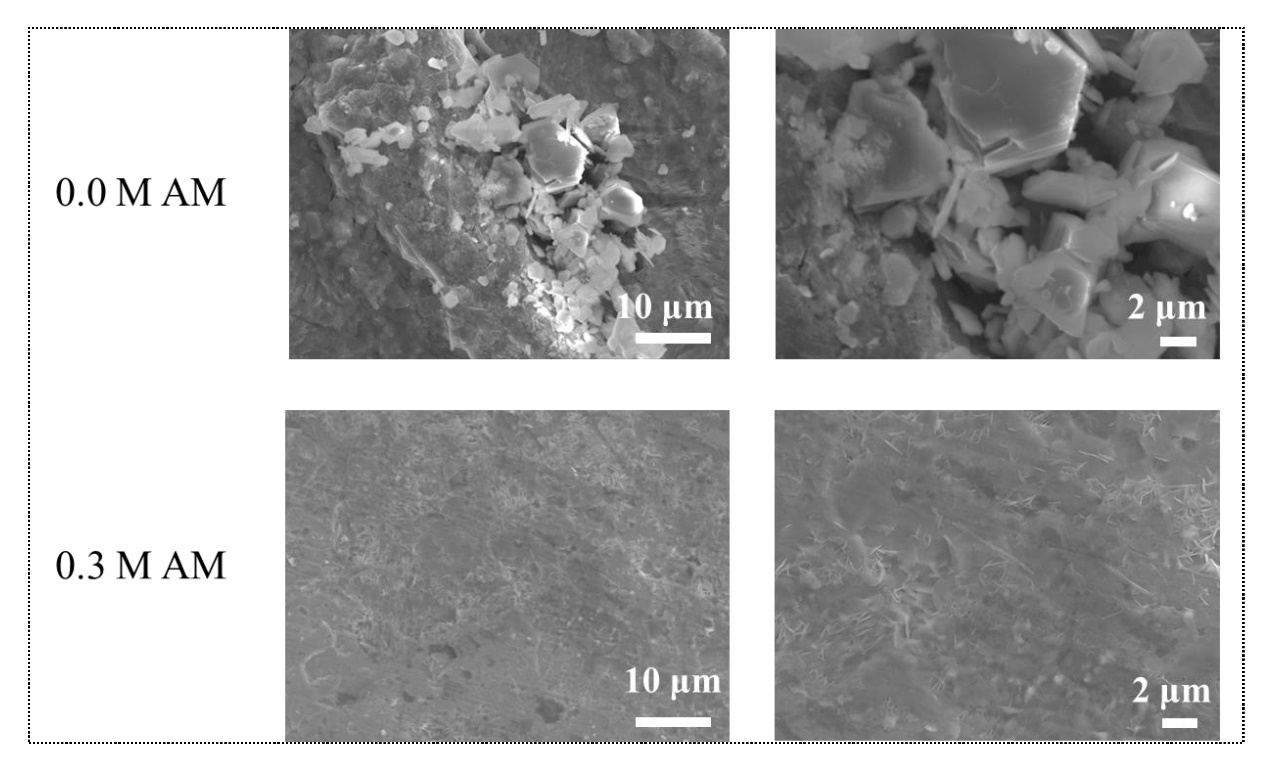


**Fig. S15** SEM images of the Zn anode surface in Zn//Zn symmetric cells after 300 h of cycling at 1 mA cm^-2^ and 1 mAh cm^-2^


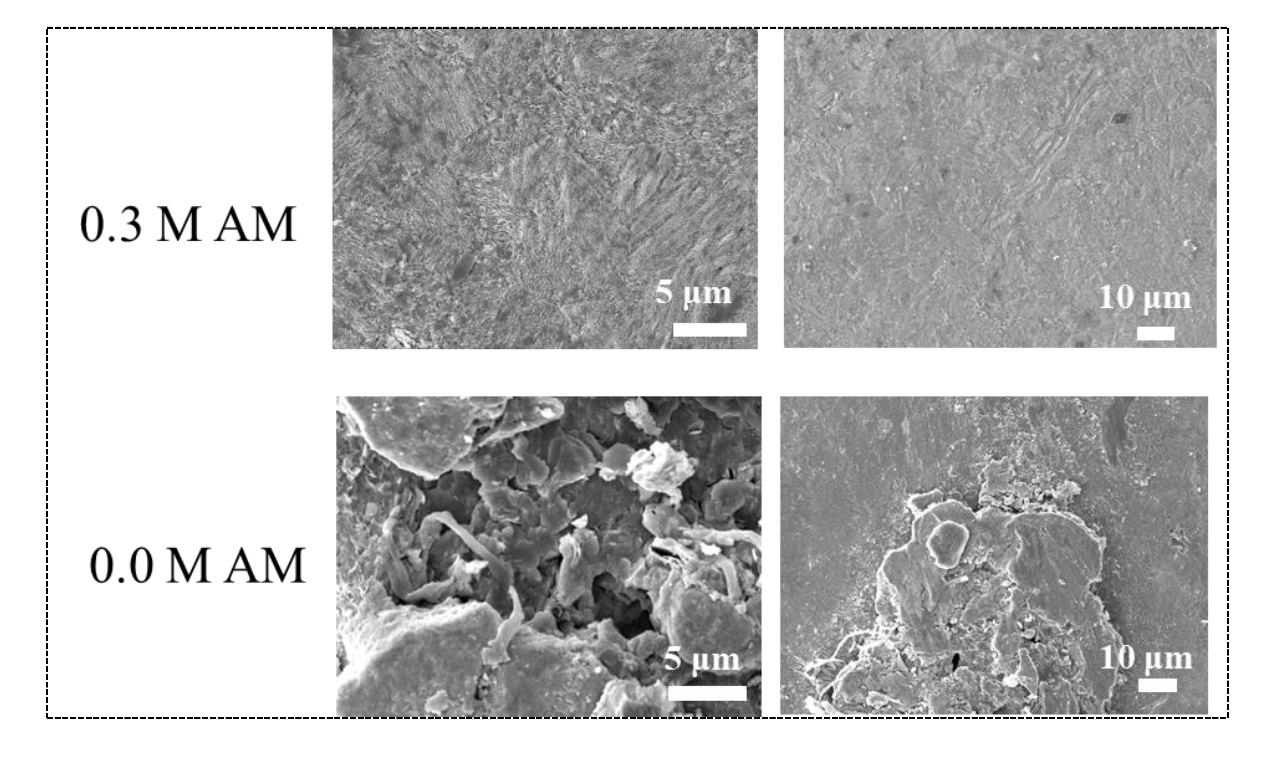


**Fig. S16** SEM images of the zinc anode surfaces in Zn//Zn symmetric cells after 100 h of cycling at 5 mA cm⁻² and 2.5 mAh cm⁻² in different electrolytes.cm^−2^ in different electrolytes

**
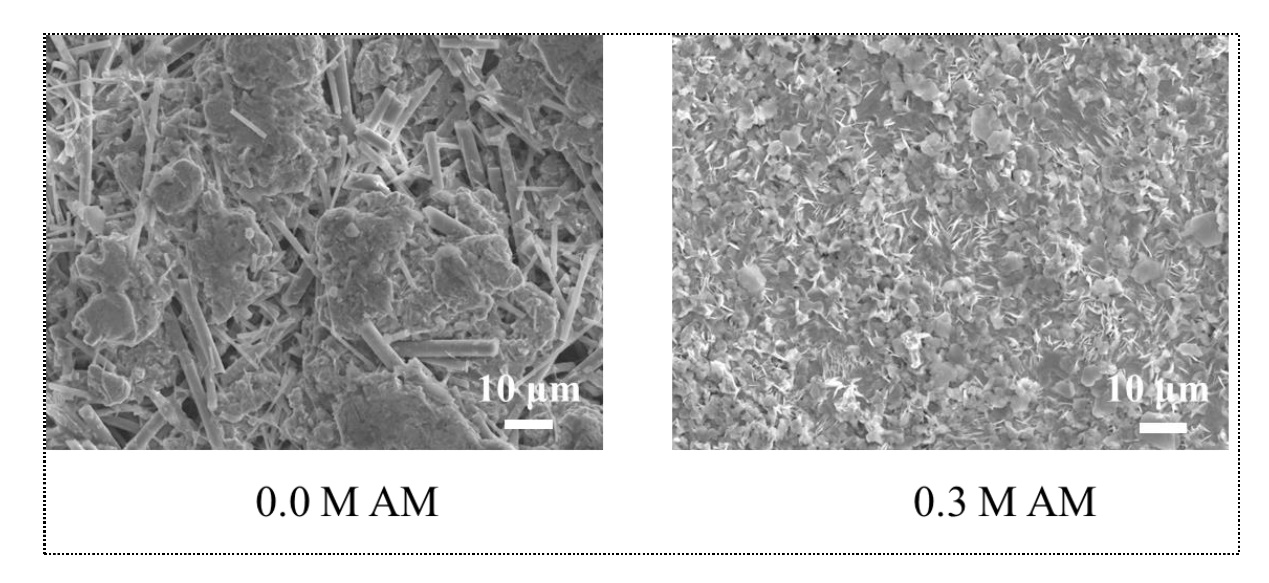
**

**Fig. S17** SEM images of the Zn anode surfaces in Zn//Zn symmetric cells after 50h at 10 mA cm⁻² and 10 mAh cm⁻² in different electrolytes


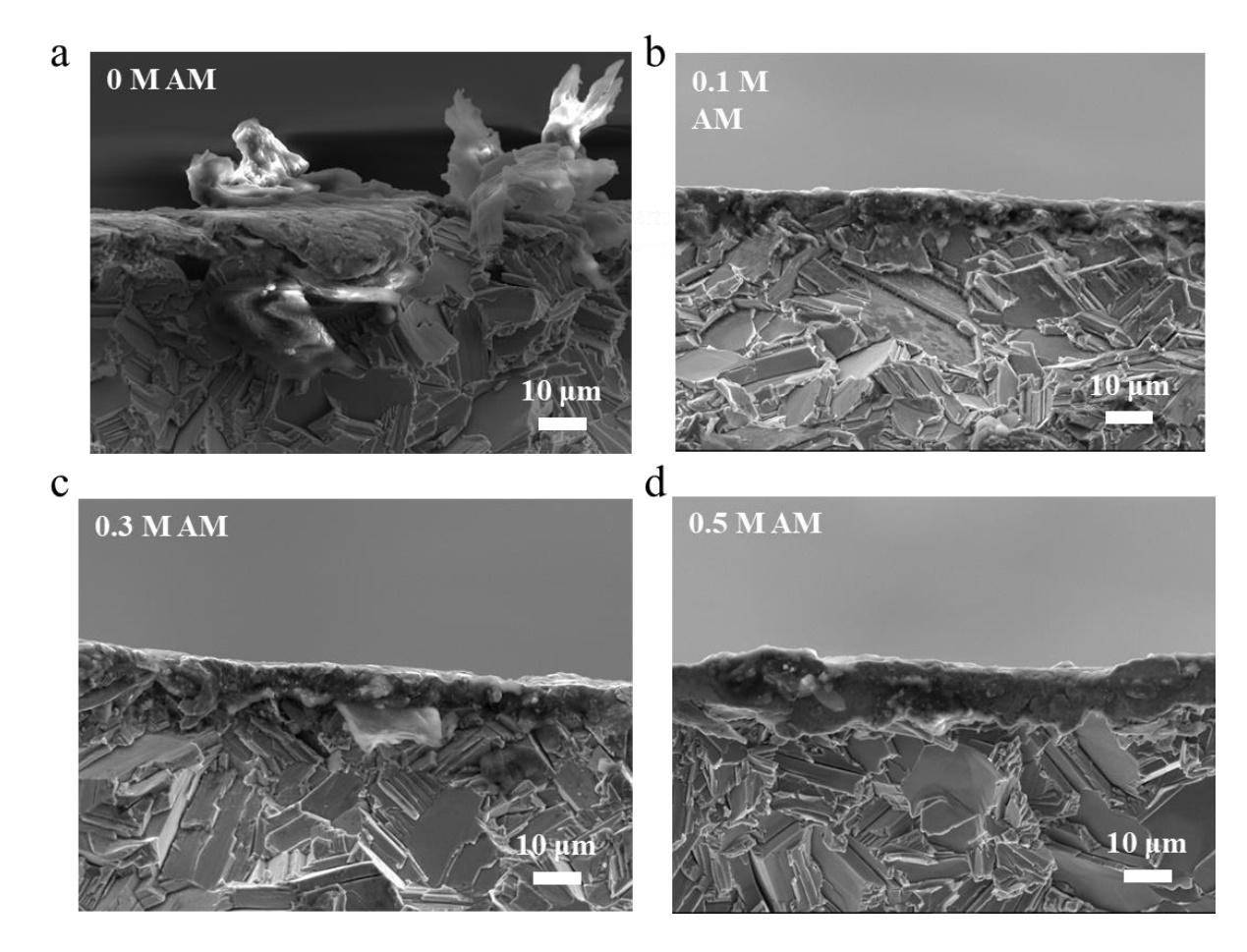


**Fig. S18** Cross-sectional SEM images of Zn anodes from Zn//Zn symmetric cells after 200 hours of cycling at 1 mA cm^-2^ and 1 mAh cm^-2^ in four different electrolytes: a 0 M AM (no HNI), **b** 0.1 M AM (HNI with a thickness of ~ 3 μm), **c** 0.3 M AM (HNI with a thickness of ~ 5 μm) and **d** 0.5 M AM (HNI with a thickness of ~ 10 μm)


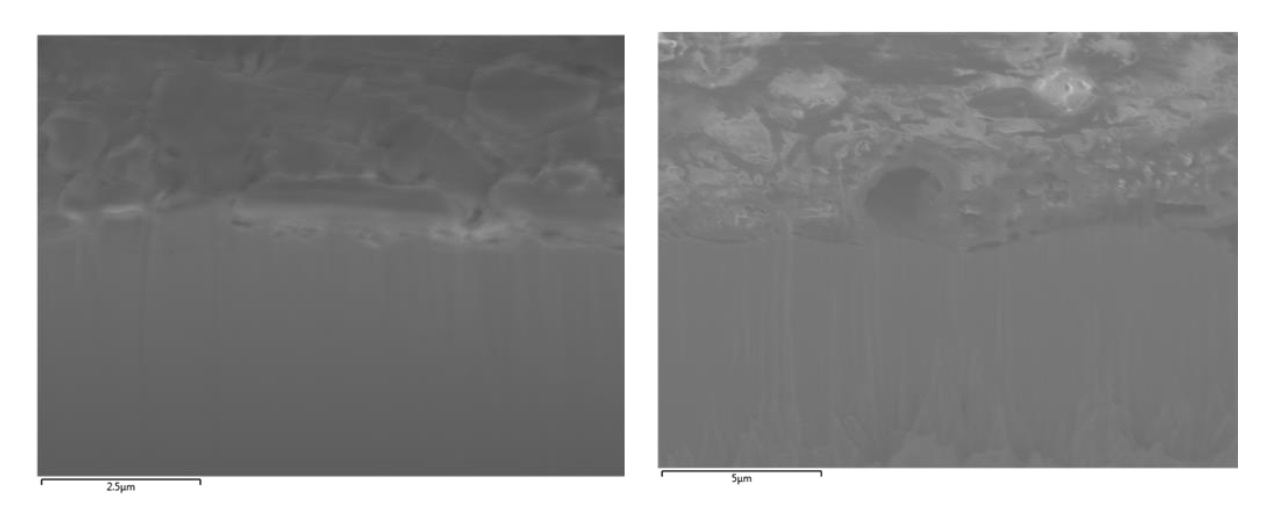


**Fig. S19** FIB cross-sectional image of the zinc anode from a Zn//Zn symmetric cell after 100 hours of cycling at 5 mA cm^-2^ and 2.5 mAh cm^-2^ in the 0.3 M AM electrolyte

**
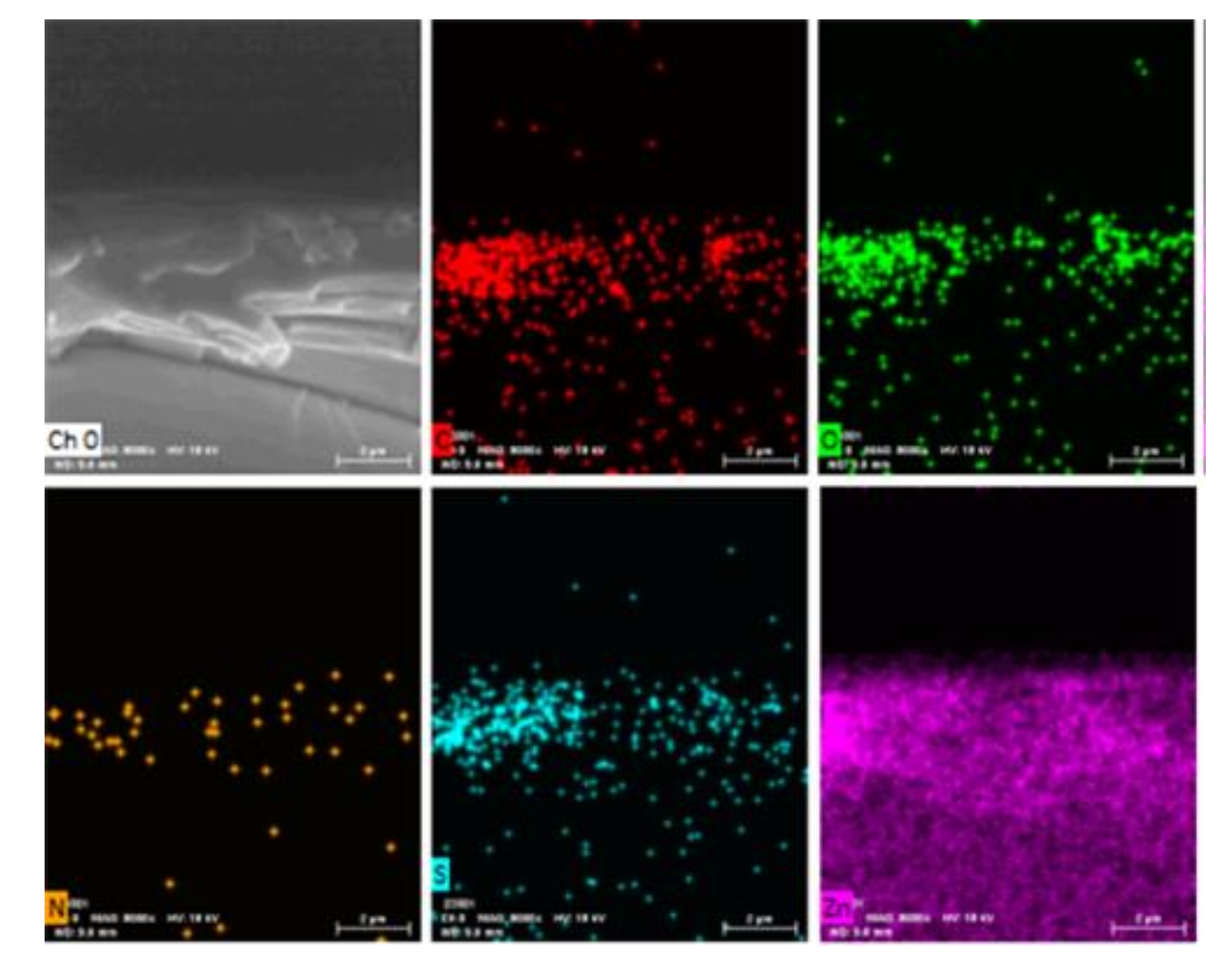
Fig. S20** EDS spectrum of the zinc anode surface in a Zn//Zn symmetric cell after 100 h of cycling at 1 mA cm^-2^ and 1 mAh cm^-2^ in 0.3 M AM electrolyte

**
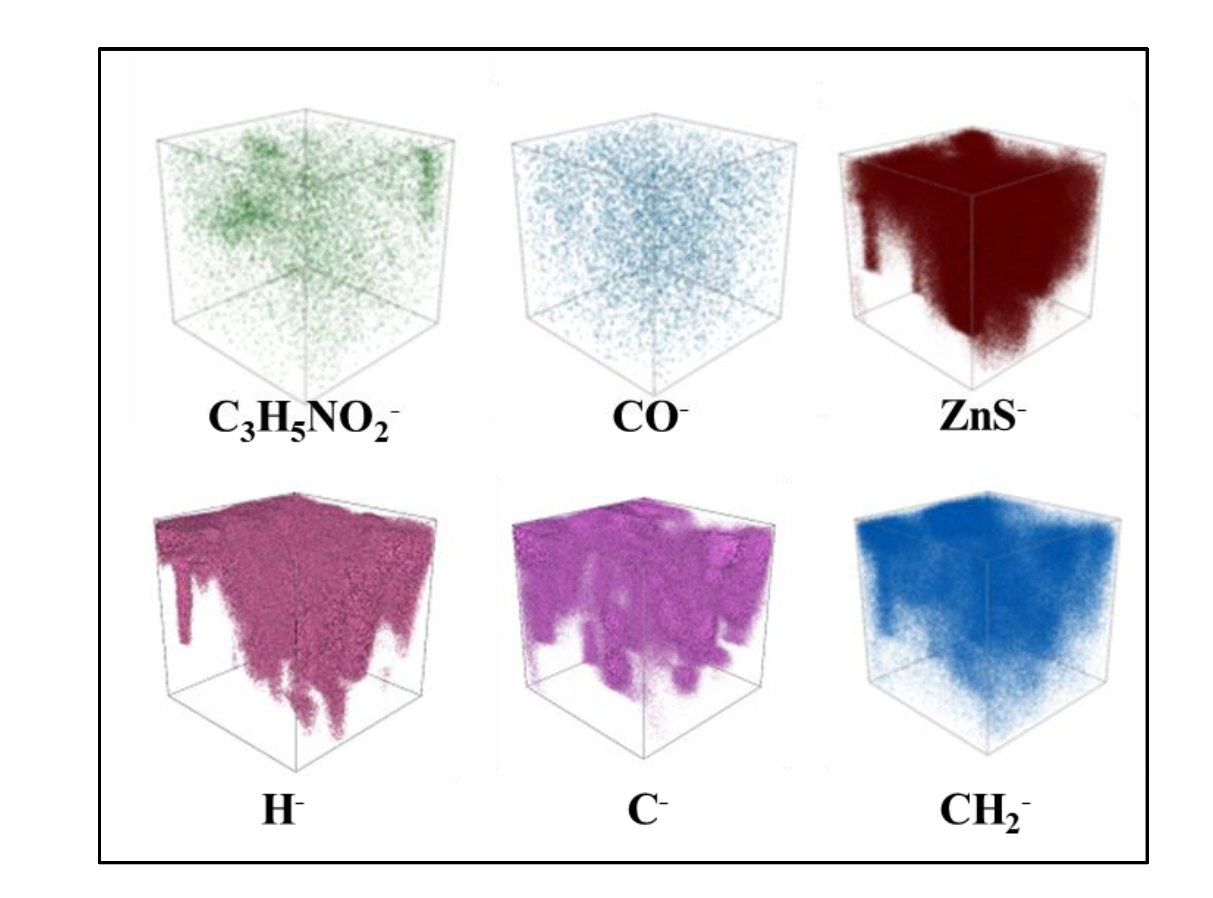
**

**Fig. S21** Three-dimensional rendering of TOF-SIMS intensity under 4500 s sputtering in negative ion mode on the cycled Zn anode surface

**
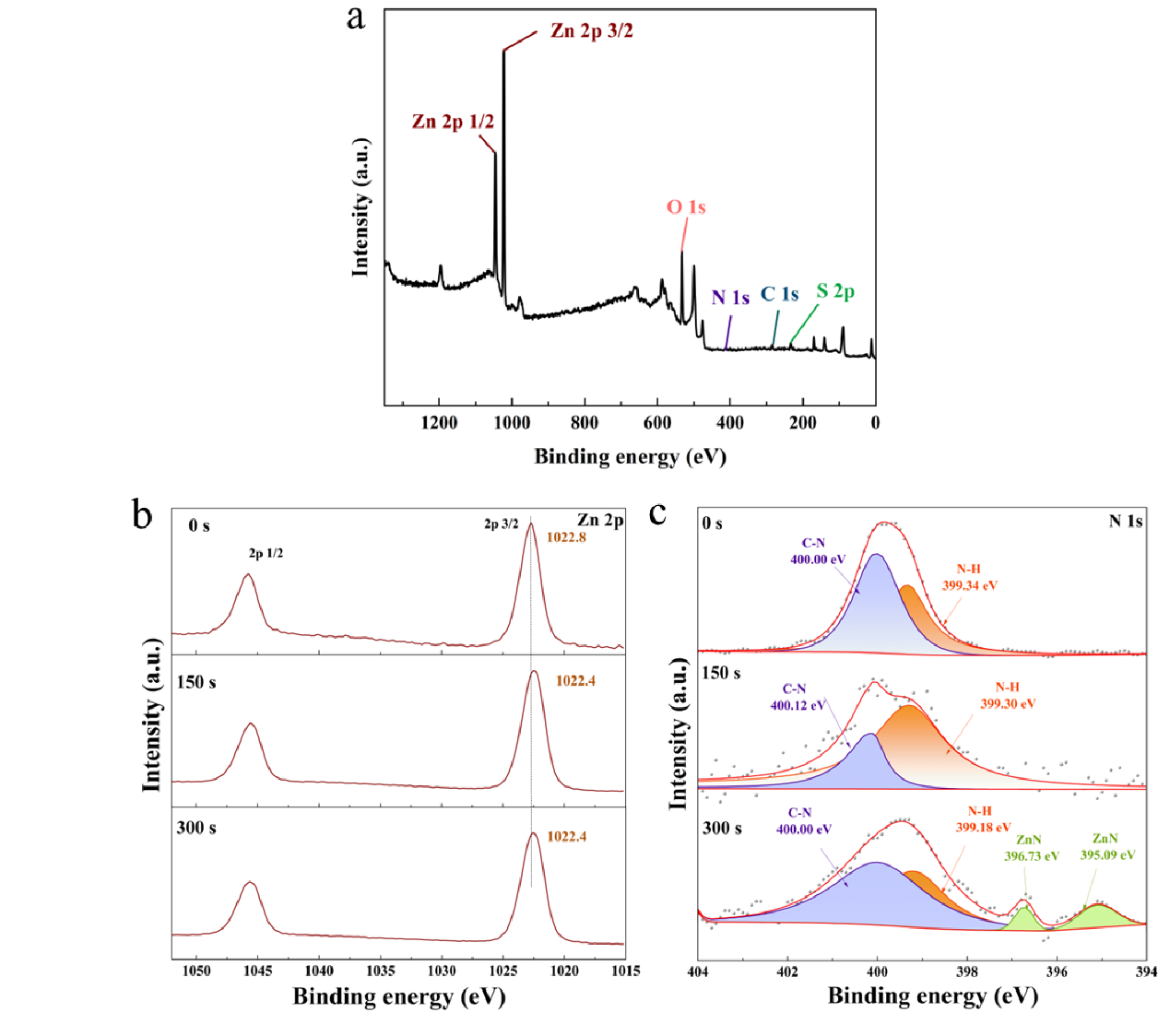
Fig. S22** XPS depth profiling with fitted peaks of the Zn anode in a Zn//Zn symmetric cell after 100 h of cycling at 1 mA cm^-2^ and 1 mAh cm^-2^ in 0.3 M AM electrolyte: **a** Survey spectrum, **b** Zn 2p and **c** N 1s


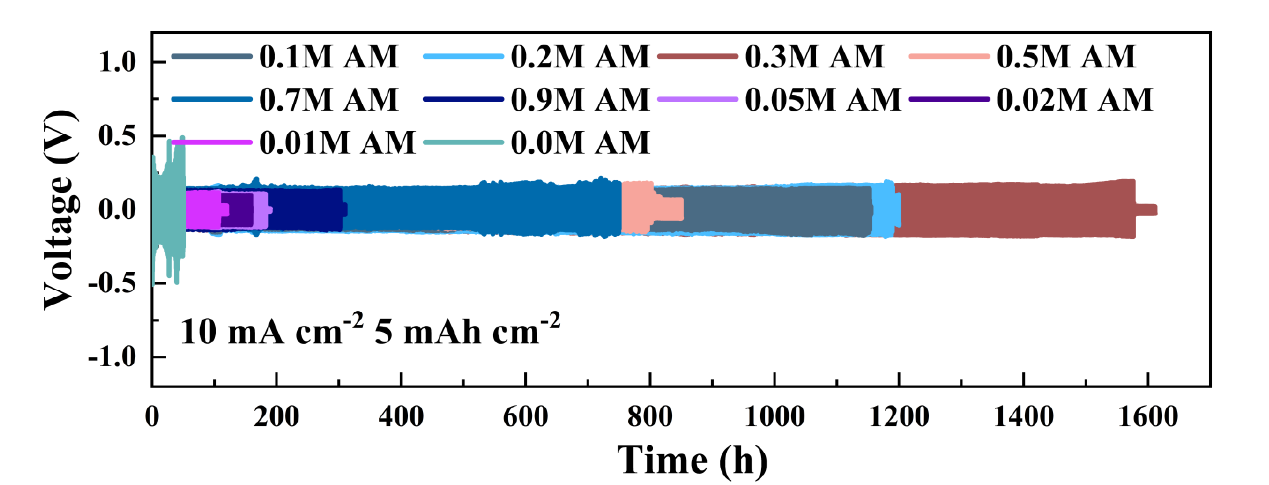


**Fig. S23** Cycling performance of packaged Zn//Zn symmetric cells at 10 mA cm^-2^ and 5 mAh cm^-2^ in electrolytes with different AM additions


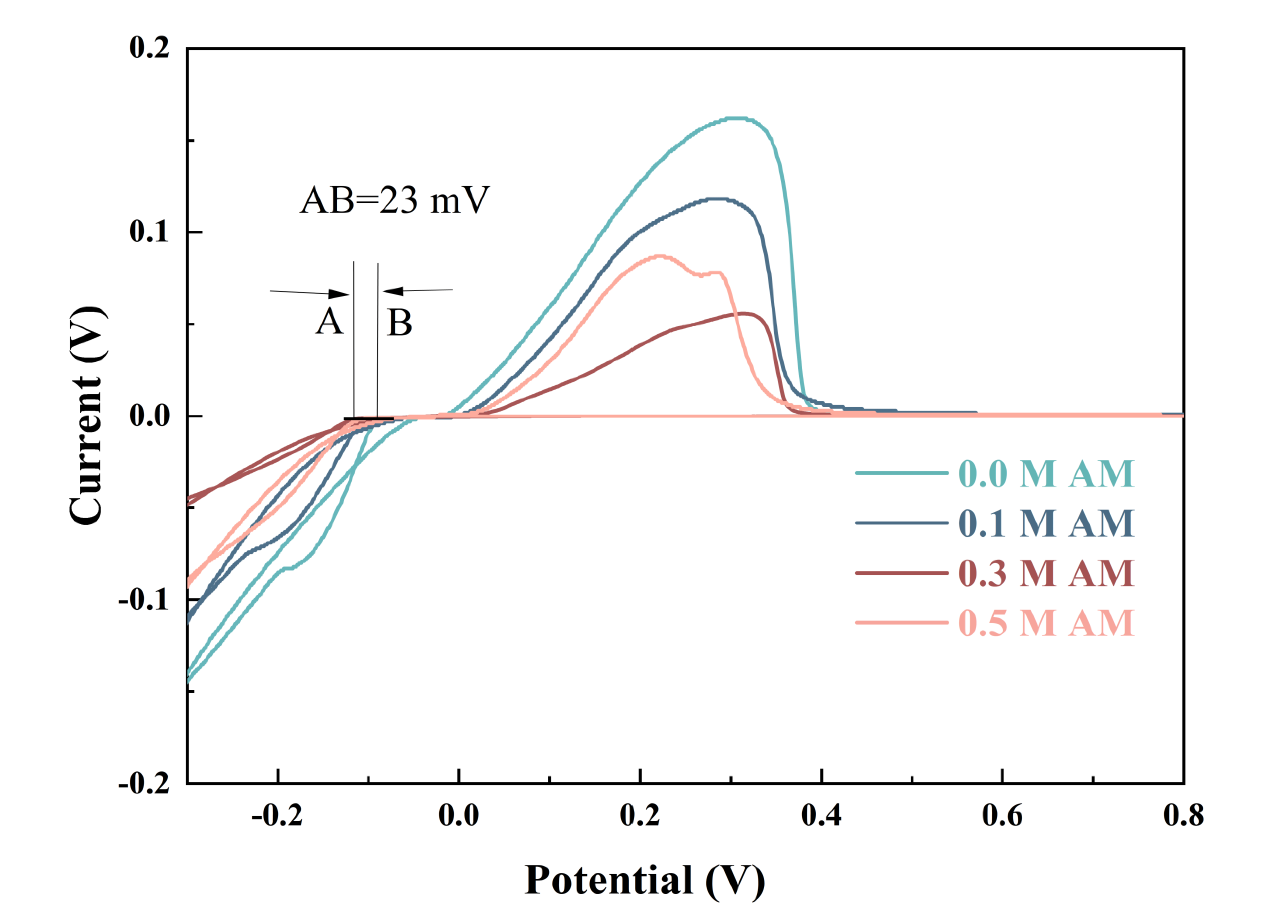


**Fig. S24** Cyclic voltammetry curves of different electrolytes


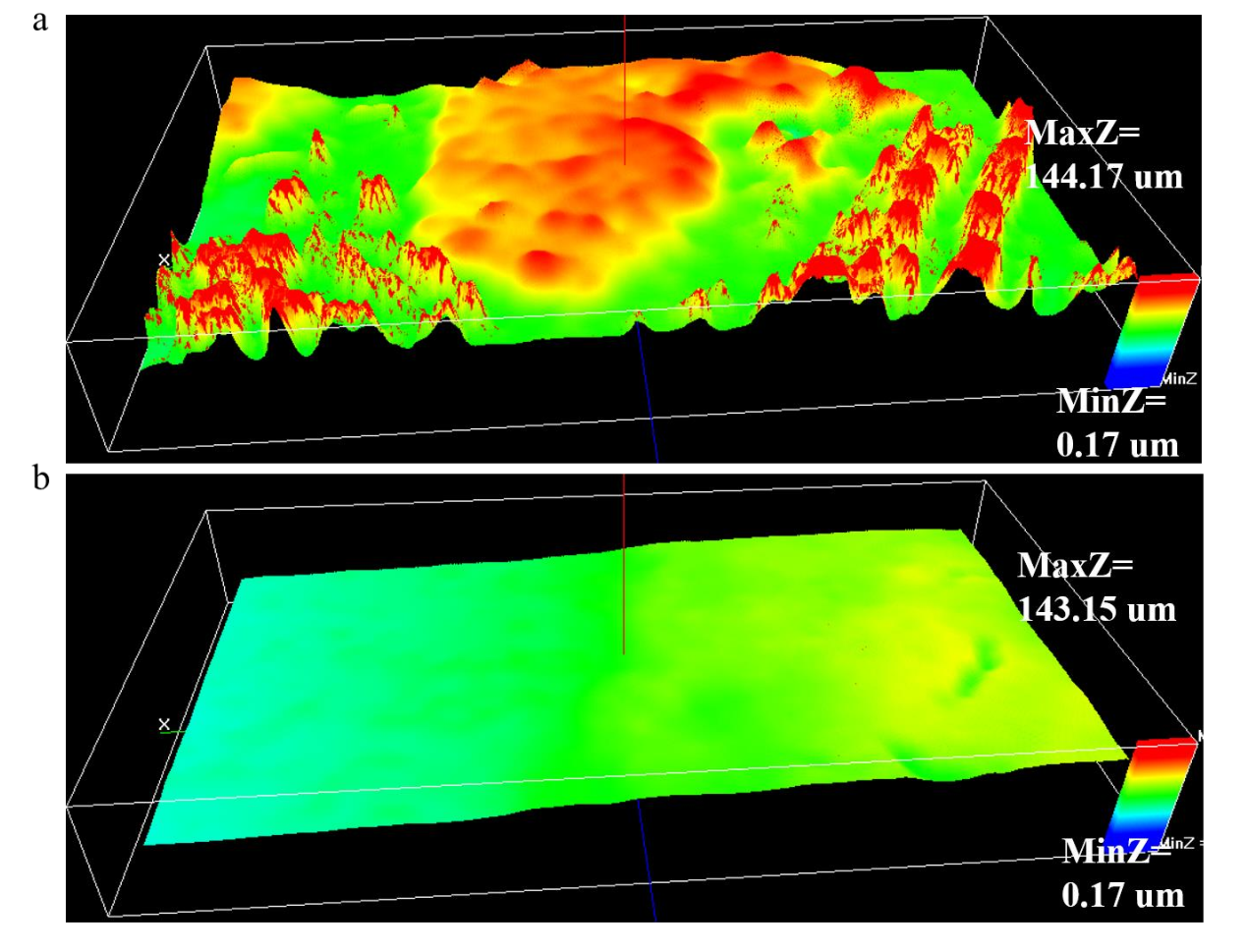


**Fig. S25** Ultra-depth microscopic images of Zn anode surfaces in Zn//Zn symmetric cells after 100 h cycling at 5 mA cm^-2^ and 2.5 mAh cm^-2^ in **a** 0 M AM electrolyte and **b** 0.3 M AM electrolyte

**
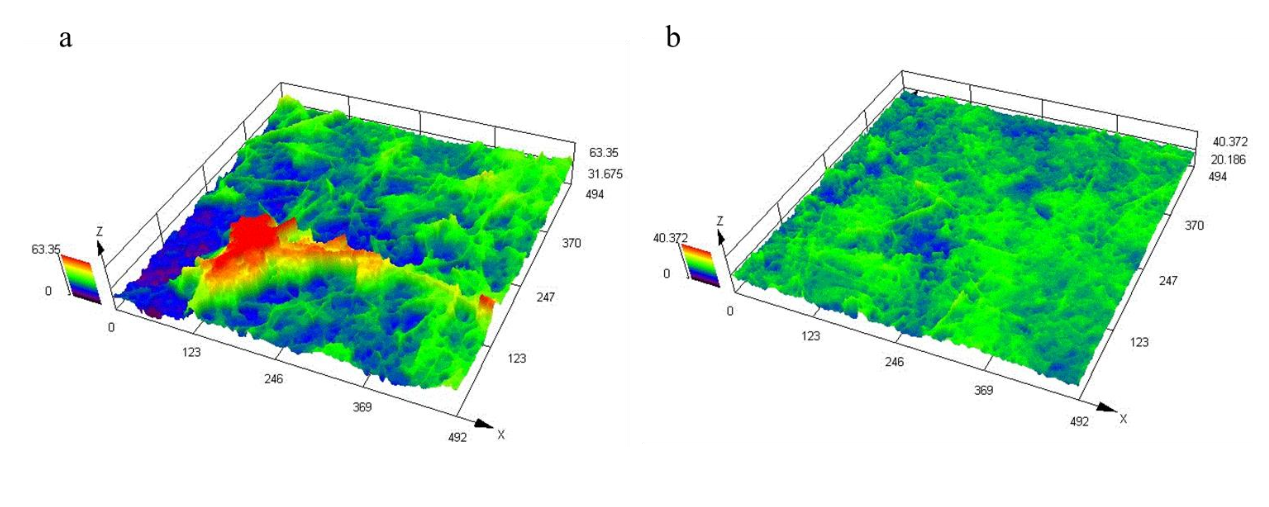
**

**Fig. S26** 3D-CLSM images of Zn anode surfaces in Zn//Zn symmetric cells after 100 h of cycling at 5 mA cm^-2^ and 2.5 mAh cm^-2^ in different electrolytes: **a** 0.0 M AM and **b** 0.3 M AM


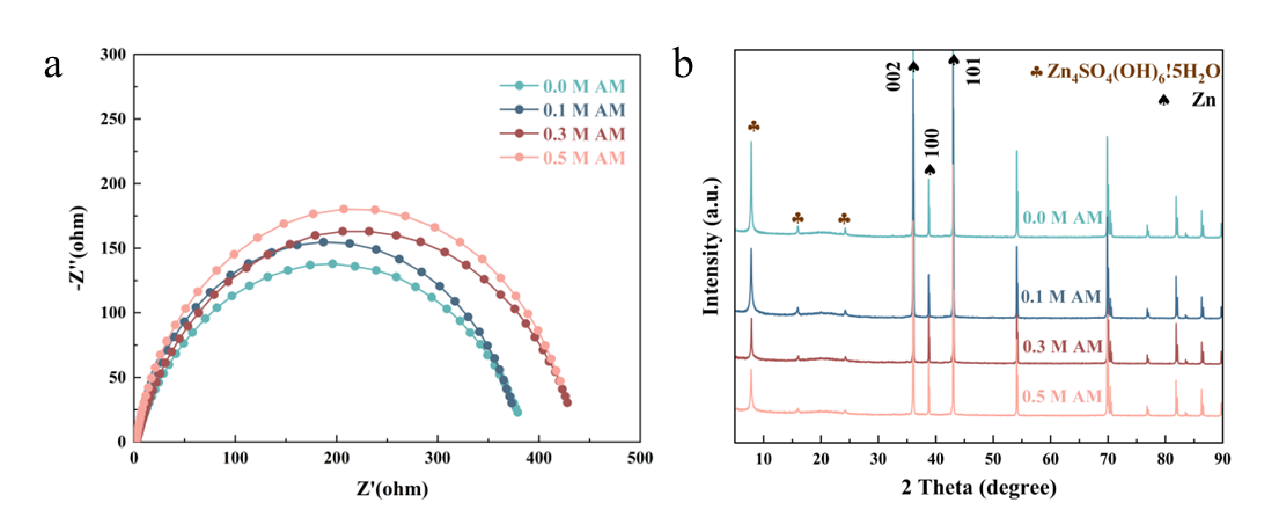


**Fig. S27 a** EIS of Zn//Ti asymmetric cells of different electrolytes. **b** XRD after 100 h of cycling in different electrolytes


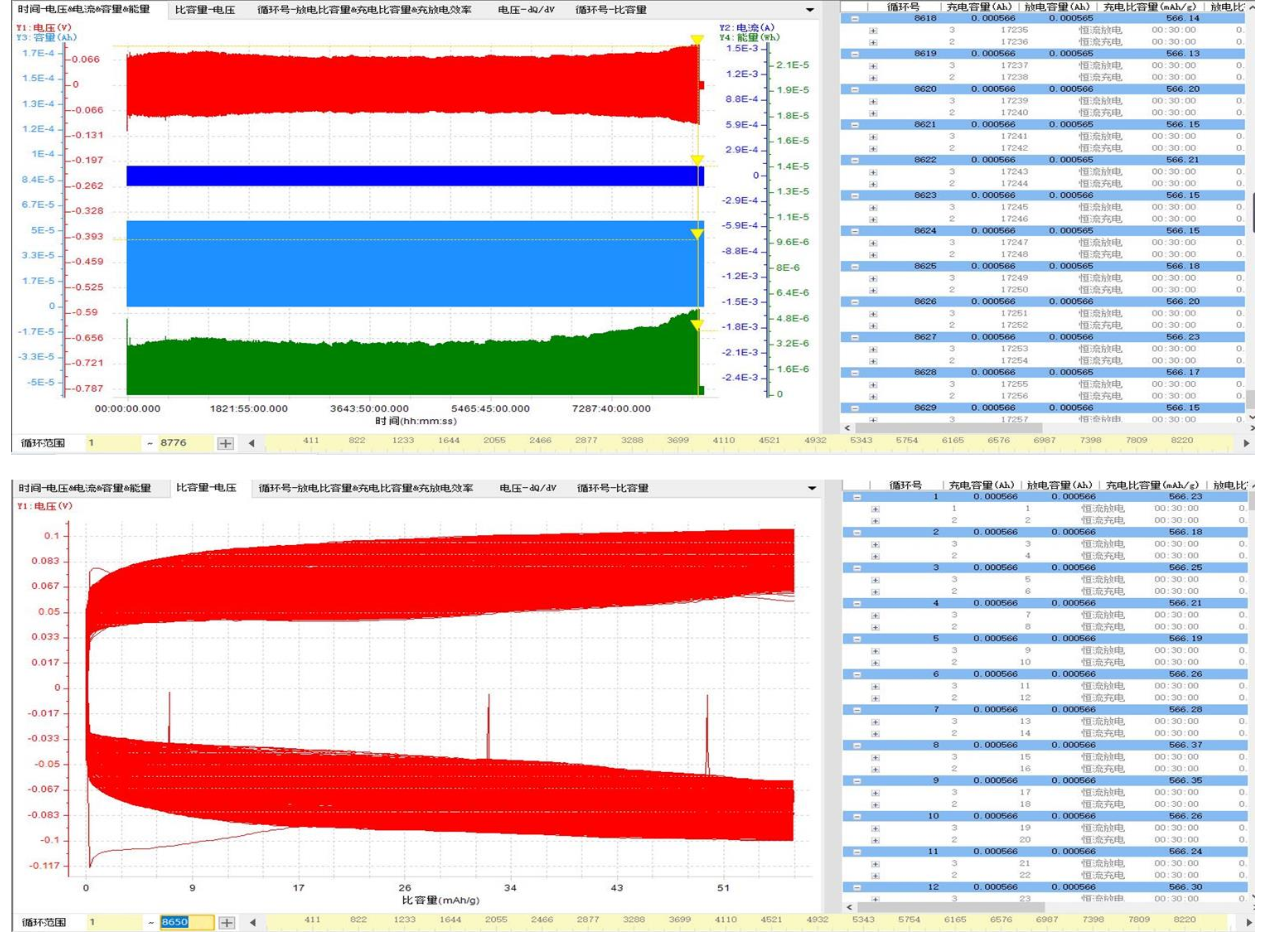


**Fig. S28** Original data screenshot from the Neware battery testing system. The complete testing interface for the Zn//Zn symmetric cell with 0.3 M AM electrolyte cycled at 1 mA cm^-2^ and 0.5 mAh cm^-2^, showing 8,650 hours of stable operation. The screenshot includes test duration, current density, areal capacity, and voltage profiles, providing direct and unambiguous evidence of data authenticity.


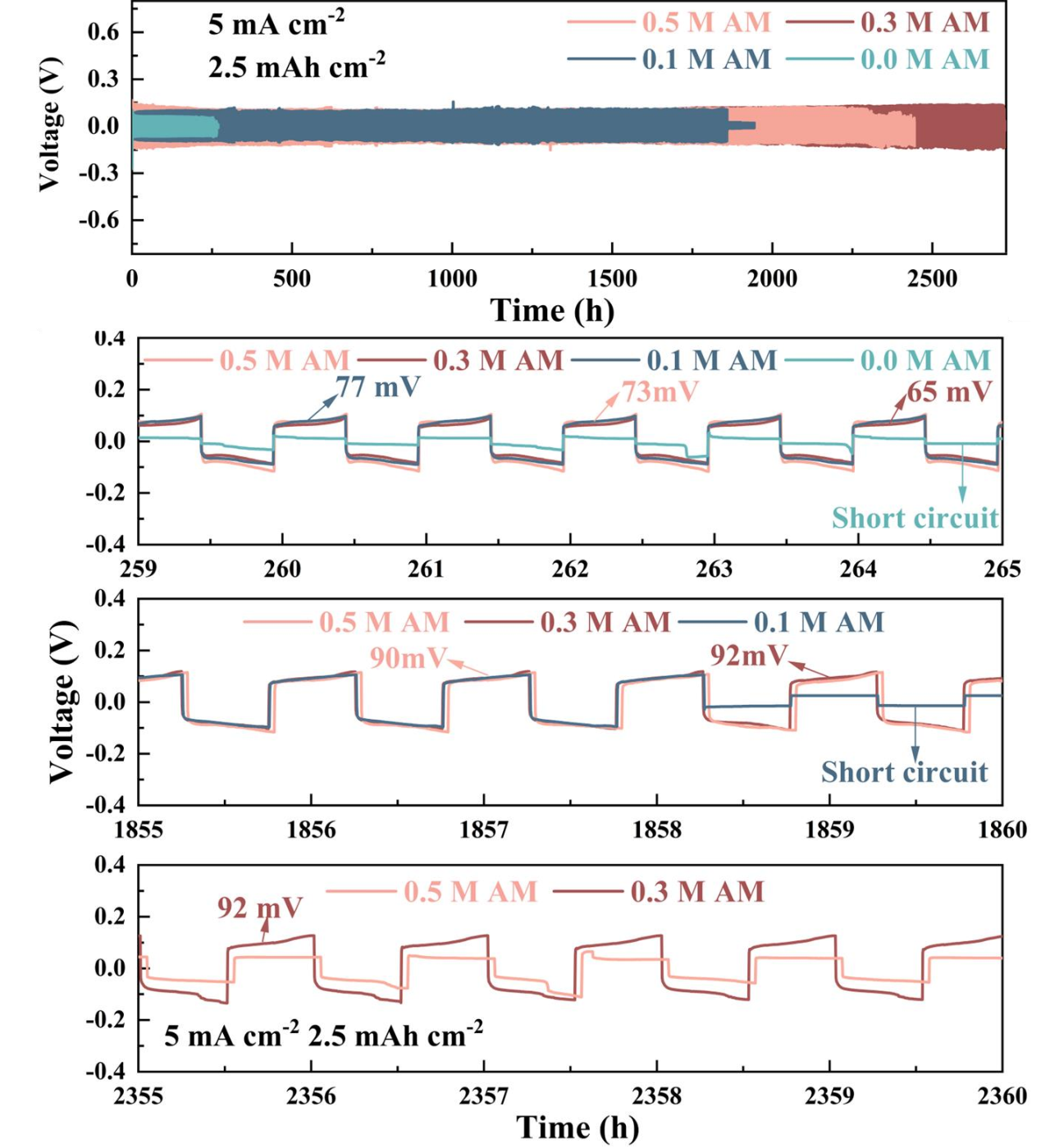


**Fig. S29** Cycling performance of Zn//Zn symmetric cells with different AM concentrations under 5 mA cm^-2^ and 2.5 mAh cm^-2^


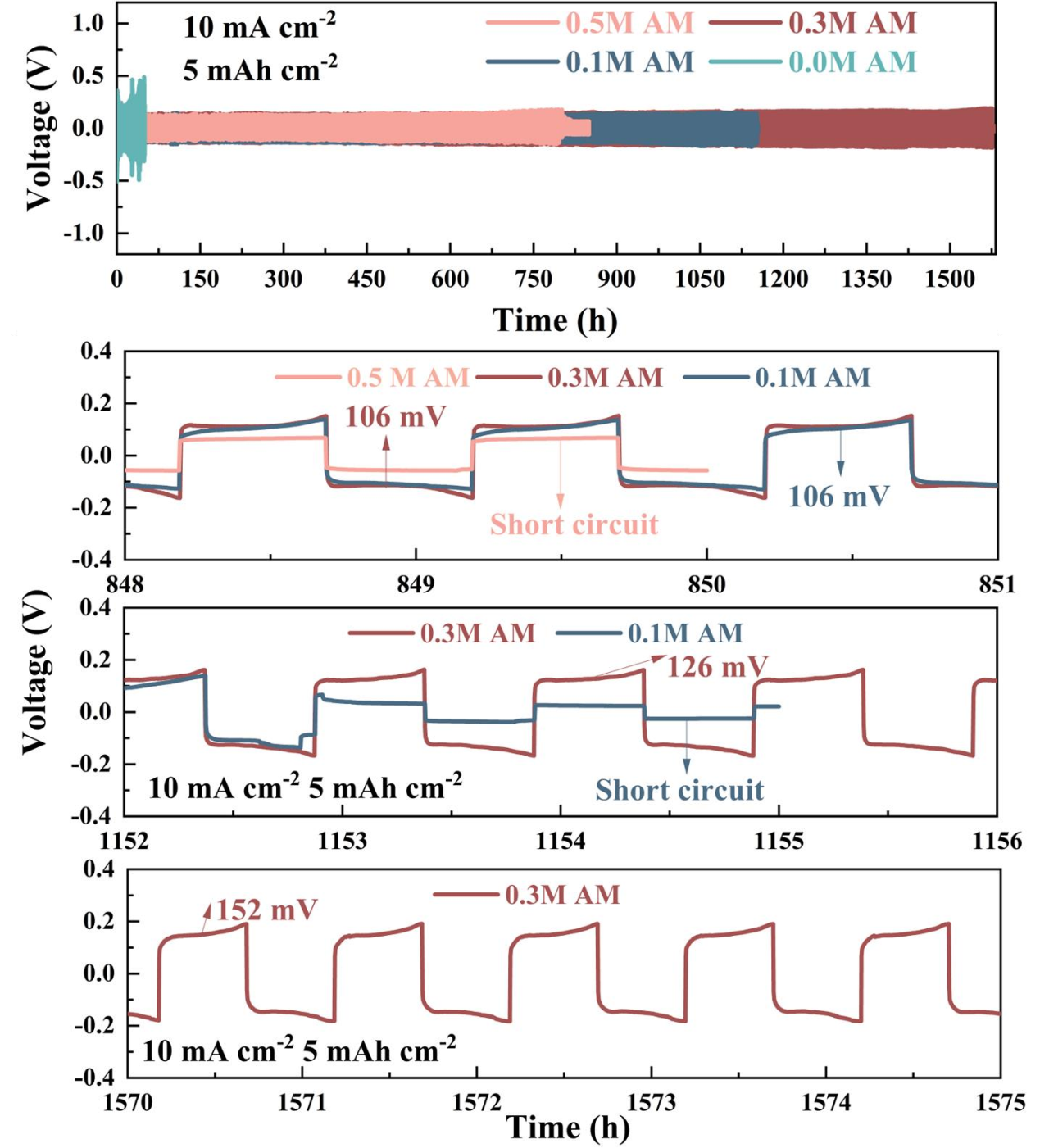


**Fig. S30** Cycling performance of Zn//Zn symmetric cells with different AM concentrations under 10 mA cm^-2^ and 5 mAh cm^-2^


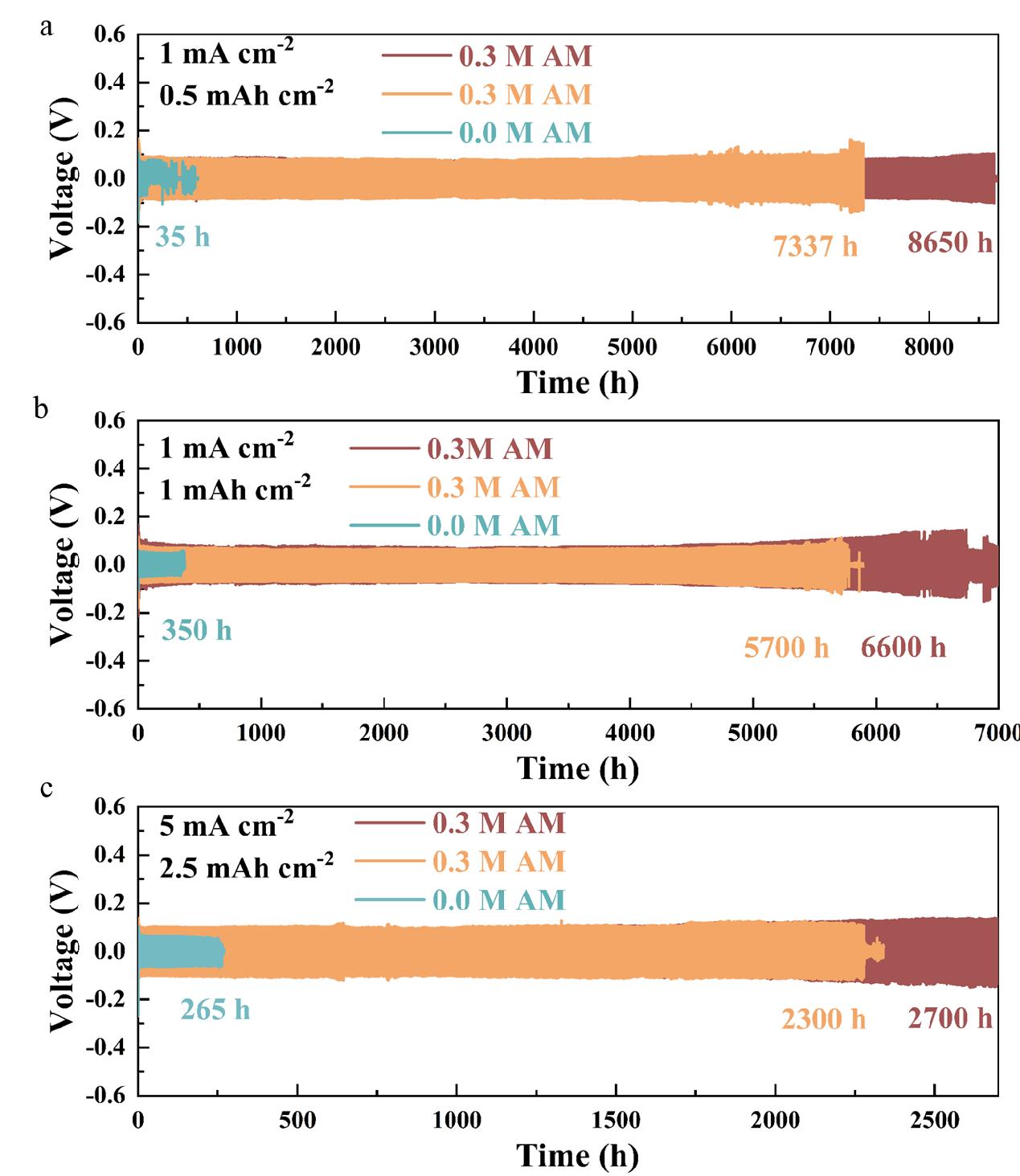


**Fig. S31** Long-term cycling voltage curves of Zn//Zn symmetric cells in electrolytes with/without AM: **a** 5 mA cm^-2^ and 2.5 mAh cm^-2^. **b** 5 mA cm^-2^ and 2.5 mAh cm^-2^. **c** 5 mA cm^-2^ and 2.5 mAh cm^-2^


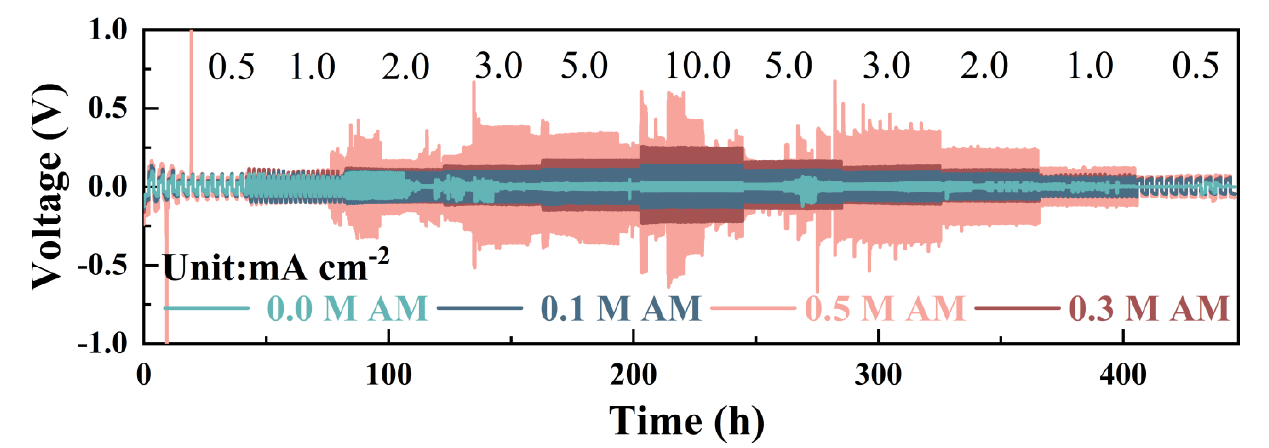


**Fig. S32** Rate performance of Zn//Zn symmetric cells with electrolytes of different AM concentrations


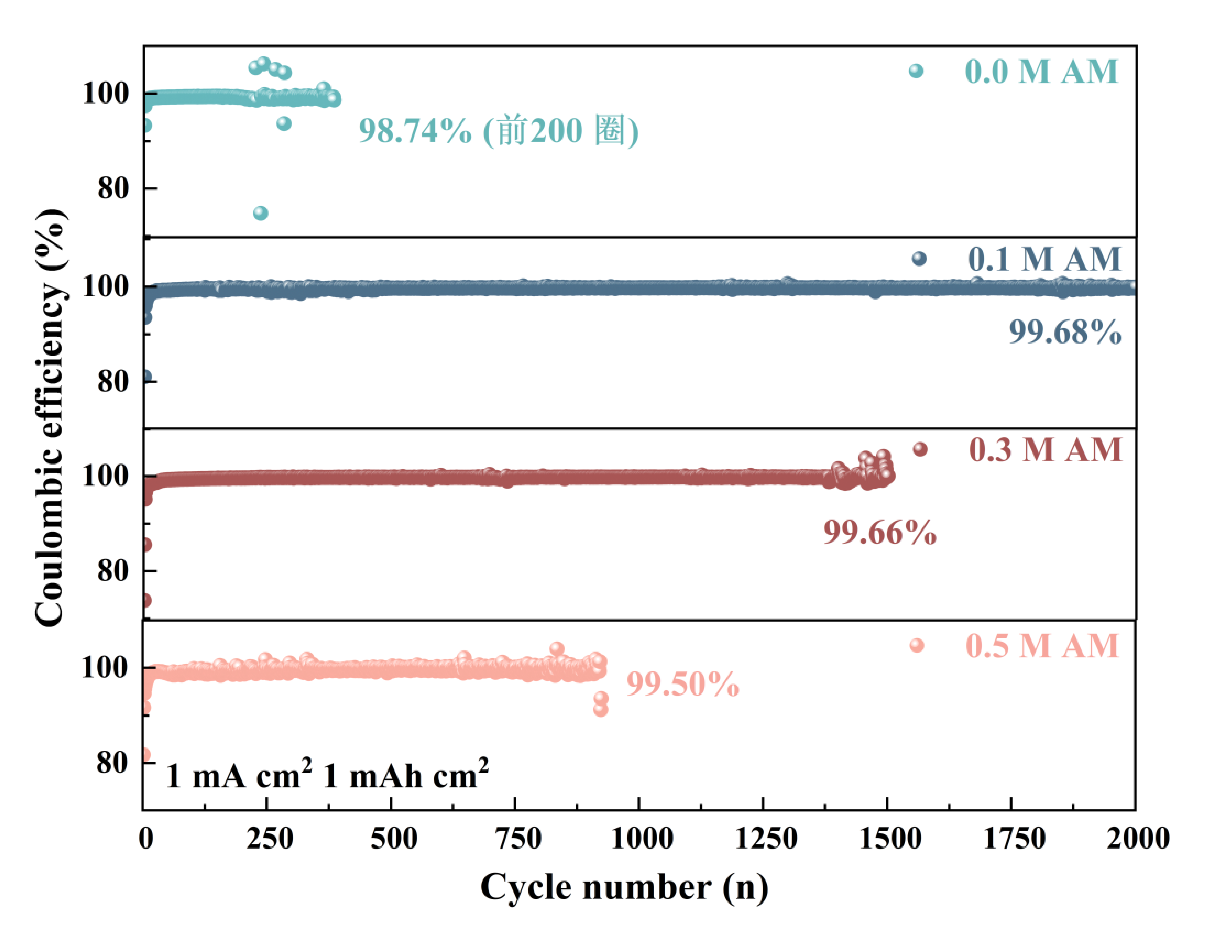


**Fig. S33** Coulombic efficiency of Zn//Cu asymmetric cells in electrolytes with different AM concentrations at 1 mAh cm^-2^


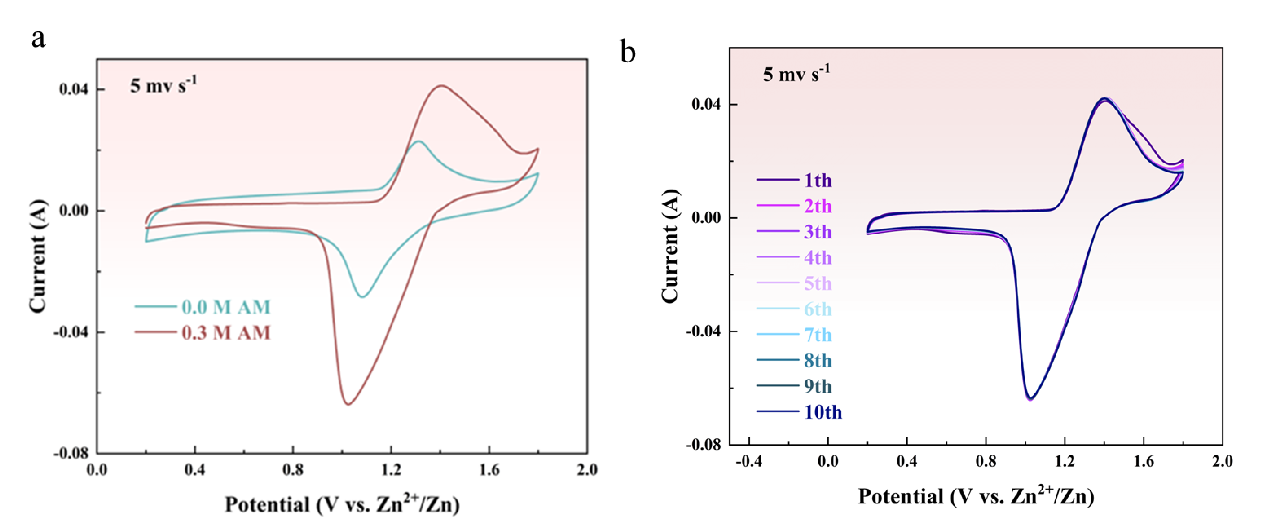


**Fig. S34** **a** CV curves of the Zn//I_2_ full cell in 0.0 M AM and 0.3 M AM electrolytes. **b** CV curves of the Zn//I_2_ full cell in 0.3 M AM electrolyte at different cycle numbers

**Supplementary** **References**

1. Y. Ding, X. Zhang, T. Wang, B. Lu, Z. Zeng et al., A dynamic electrostatic shielding layer toward highly reversible zn metal anode. Energy Storage Mater. **62**, 102949 (2023). [https://doi.org/10.1016/j.ensm.2023.102949](https://doi.org/https://doi.org/10.1016/j.ensm.2023.102949)
2. B. Hu, T. Chen, Y. Wang, X. Qian, Q. Zhang et al., Reconfiguring the electrolyte network structure with bio-inspired cryoprotective additive for low-temperature aqueous zinc batteries. Adv. Energy Mater. **14**(31), 2401470 (2024). [https://doi.org/10.1002/aenm.202401470](https://doi.org/https://doi.org/10.1002/aenm.202401470)
3. H. Qin, W. Kuang, N. Hu, X. Zhong, D. Huang et al., Building metal-molecule interface towards stable and reversible zn metal anodes for aqueous rechargeable zinc batteries. Adv. Funct. Mater. **32**(47), 2206695 (2022). [https://doi.org/10.1002/adfm.202206695](https://doi.org/https://doi.org/10.1002/adfm.202206695)
4. Y. Zhang, S. Shen, K. Xi, P. Li, Z. Kang et al., Suppressed dissolution of fluorine-rich sei enables highly reversible zinc metal anode for stable aqueous zinc-ion batteries. Angew. Chem. Int. Ed. **63**(32), e202407067 (2024). [https://doi.org/10.1002/anie.202407067](https://doi.org/https://doi.org/10.1002/anie.202407067)
5. Q. Zong, R. Li, J. Wang, Q. Zhang, A. Pan, Tailoring the whole deposition process from hydrated zn^2+^ to zn0 for stable and reversible zn anode. Angew. Chem. Int. Ed. **63**(41), e202409957 (2024). [https://doi.org/10.1002/anie.202409957](https://doi.org/https://doi.org/10.1002/anie.202409957)
6. H. Geng, M. Cheng, B. Wang, Y. Yang, Y. Zhang et al., Electronic structure regulation of layered vanadium oxide via interlayer doping strategy toward superior high-rate and low-temperature zinc-ion batteries. Adv. Funct. Mater. **30**(6), 1907684 (2020). [https://doi.org/10.1002/adfm.201907684](https://doi.org/https://doi.org/10.1002/adfm.201907684)
7. T. Wei, X. Zhang, Y. Ren, Y. Wang, Z. Li et al., Reconstructing anode/electrolyte interface and solvation structure towards high stable zinc anode. Chem. Eng. J. **457**, 141272 (2023). [https://doi.org/10.1016/j.cej.2023.141272](https://doi.org/https://doi.org/10.1016/j.cej.2023.141272)
